# Materials for the Spine: Anatomy, Problems, and Solutions

**DOI:** 10.3390/ma12020253

**Published:** 2019-01-14

**Authors:** Brody A. Frost, Sandra Camarero-Espinosa, E. Johan Foster

**Affiliations:** 1 Department of Materials Science and Engineering, Macromolecules Innovation Institute, Virginia Tech, Blacksburg, VA 24061, USA; bfrost12@vt.edu; 2 Complex Tissue Regeneration Department, MERLN Institute for Technology-inspired Regenerative Medicine, Maastricht University, P.O. Box 616, 6200MD Maastricht, The Netherlands

**Keywords:** spinal anatomy, intervertebral disc, degenerative disc disease, herniated disc, spinal fusion, total disc replacement, tissue engineering

## Abstract

Disc degeneration affects 12% to 35% of a given population, based on genetics, age, gender, and other environmental factors, and usually occurs in the lumbar spine due to heavier loads and more strenuous motions. Degeneration of the extracellular matrix (ECM) within reduces mechanical integrity, shock absorption, and swelling capabilities of the intervertebral disc. When severe enough, the disc can bulge and eventually herniate, leading to pressure build up on the spinal cord. This can cause immense lower back pain in individuals, leading to total medical costs exceeding $100 billion. Current treatment options include both invasive and noninvasive methods, with spinal fusion surgery and total disc replacement (TDR) being the most common invasive procedures. Although these treatments cause pain relief for the majority of patients, multiple challenges arise for each. Therefore, newer tissue engineering methods are being researched to solve the ever-growing problem. This review spans the anatomy of the spine, with an emphasis on the functions and biological aspects of the intervertebral discs, as well as the problems, associated solutions, and future research in the field.

## 1. Human Spinal Anatomy

The spine, or vertebral column, is a bony structure that houses the spinal cord and extends the length of the back, connecting the head to the pelvis [1].

**Figure 1:**
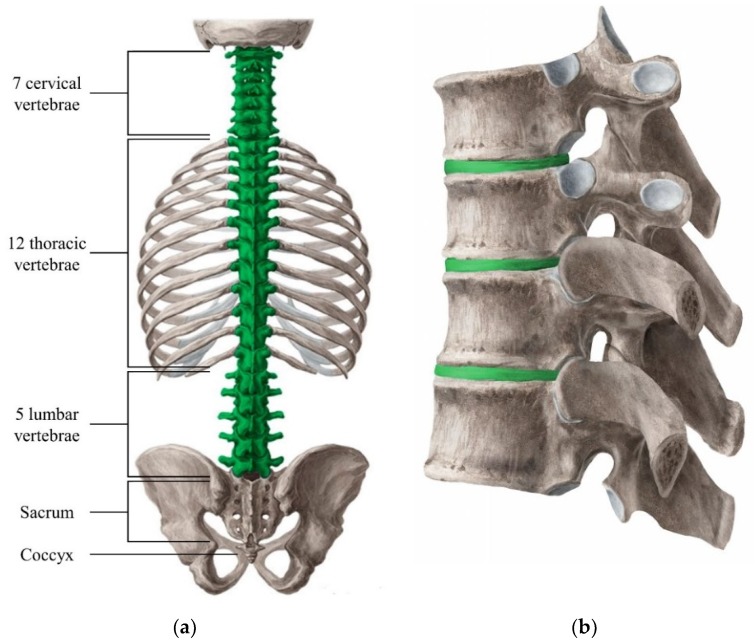
Overview of the vertebral column with each specific section labeled for clarification (**a**). The green highlighted section refers to the part of the spine that contain individual vertebrae, as well as intervertebral discs (IVD). The structure of the vertebrae and IVD (green highlighted) have been added for better visualization (**b**) [4].

The most important function of the spine is to protect the spinal cord, which is the nerve supply for the entire body originating in the brain [1]. Along with this major function, others include supporting the mass of the body, withstanding external forces, and allowing for mobility and flexibility while dissipating energy and protecting against impact. The spine is connected to the muscles and ligaments of the trunk for postural control and spinal stability [2]. It can be separated into five distinct sections, the cervical spine, the thoracic spine, the lumbar spine, the sacrum, and the coccyx, all of which are comprised of independent bony vertebrae and intervertebral discs [3], Figure 1. To describe the differences between the spinal column sections, each one has been further discussed.

### 1.1. Cervical Spine

The cervical section of the spine consists of seven vertebrae (C1–C7) and six intervertebral discs, and extends from the base of the skull to the top of the trunk, where the thoracic vertebrae and rib cage start [3] Figure 1. The cervical spine’s major functions include supporting and cushioning loads to the head/neck while allowing for rotation, and protecting the spinal cord extending from the brain [5].

Of these seven vertebrae, the atlas (C1) and the axis (C2) are among the most important for rotation and movement of the head [6]. The atlas is the only cervical vertebra that does not contain a vertebral body, but instead has a more ring-like structure for cradling the skull at the occipital bone, creating the atlanto-occipital joint. This joint in particular makes up for about 50% of the head’s flexion and extension range of motion [5,6,7]. The axis contains a large bony protrusion (the odontoid process) that extends from the body, superiorly, into a facet on the ring-shaped atlas, forming the atlanto-axial joint [5,6]. This connection allows the head and atlas to rotate from side to side as one unit, and accounts for about 50% of the neck’s rotation, as well as having the function of transferring the weight of the head through the rest of the cervical spine [5,6,7]. The rest of the vertebrae (C3–C7), have significantly reduced mobility, however are mainly used as support for the weight bearing of the head and other loads applied onto the neck. 

The cervical spine protects both the efferent and afferent nerves that stem from the spinal cord and, if damaged, can lead to dramatic effects on the nervous system eventually affecting the patient’s daily activity, and even causing a potential paralysis [8]. The cushioning and support of loads by the intervertebral discs are crucial to the longevity of vertebrae, and therefore, the nerves, since they run through the same joint separation [9]. However, because of the extensive movement that occurs in the cervical spine, the intervertebral discs go through drastic changes in stresses and strains causing them to be much more susceptible to injury, which can cause damage to or impingements on these nerves [9]. This can lead to feelings of weakness, numbness, tingling, and potentially loss of feeling. 

### 1.2. Thoracic Spine

The thoracic section of the spine consists of twelve vertebrae (T1–T12) and twelve intervertebral discs, and extends from the bottom of the cervical spine to the beginning of the lumbar spine [3], Figure 1. The thoracic spine’s major functions include heavy load bearing and protection of the spinal cord, supporting posture and stability throughout the trunk, and connection of the rib cage that houses and protects vital organs, such as the heart and lungs [10]. 

This connection poses a significant decrease in mobility, as compared to the cervical spine section, and a greater stability and support of the entire trunk, usually leading to fewer cases of disc degeneration [10,11]. The vertebrae that make up the thoracic spine have body sizes (thickness, width, and depth) that drastically increases descending from T1 to T12, corresponding to an increased load bearing that is transferred from the vertebra above [12]. All other features stay relatively the same throughout, except for the T11 and T12 vertebrae, in which no ribs are connected. Along with this change towards the end of the thoracic spine, the T12 plays an interfacial role and has distinct thoracic characteristics superiorly and lumbar characteristics inferiorly for articulation with the L1 vertebra, allowing rotational movements with T11 while disallowing movements with L1 [12].

The thoracic spine contains nerves that are much less specialized per vertebrae like that of the cervical and lumbar spine, however they are no less important. The afferent and efferent nerves that stem from the spinal cord in this section power the muscles that lie around (major back, chest, and abdominal muscles) and between (intercostal muscles) the ribs [13]. The sympathetic nervous system, which stems from the entire thoracic spine and top two lumbar vertebrae and help power the intercostal muscles, is necessary for vital involuntary functions such as increasing heart rate, increasing blood pressure, controlling breathing rate, regulating body temperature, air passage dilation, decreasing gastric secretions, bladder function (bladder muscle relaxation, and storage of urine), and sexual function [13]. The thoracic spine and sacrum are the only sections of the spinal cord that these involuntary nervous systems stem from, and if impinged, can cause similar problems as discussed for the cervical spine. As mentioned previously, with these nerves passing through the same proximity as the intervertebral discs, cushioning of loads and proper weight dissipation is crucial for disc health and nerve protection, although the structural support of the ribcage makes damage to these discs much less prevalent [11].

### 1.3. Lumbar Spine

The lumbar section of the spine consists of five vertebrae (L1–L5) and five intervertebral discs, and extends from the bottom of the thoracic spine to the beginning of the sacrum, which attaches the spine to the pelvis [3], Figure 1. The lumbar spine’s major functions include heavy load bearing and protection of the spinal cord during locomotion and bending/torsion of the trunk, providing maximum stability while maintain crucial mobility of the trunk about the hips/pelvis [14].

This particular section of the spine needs to be the most resilient due to the vital functions it provides. Not only does it need to support all of the transferred weight from the previous spinal sections (virtually the entire human body), but it also needs to be able to retain its mobility under these strenuous conditions. The lumbar spine, from bending over to standing straight, can go through more than a 50° range for the average person (± 28.0° from 0° bend) [15]. As well as bending motion, rotation becomes a big factor, with each normal lumbar segment having the ability to undergo up to 7°–7.5° of rotation [16]. When weight is added to these conditions, such as bending over to pick up a backpack or a weight from the floor, an immense amount of stress and strain is induced into the lumbar spine [17]. Because of this, the vertebrae and intervertebral discs in the lumbar spine are the greatest in thickness, width, and depth [18]. The L1 vertebra starts out with a thickness, width, and depth greater than any of the cervical or thoracic vertebrae, and the trend only continues as the lumbar spine continues to descend to the L5 vertebra [18]. Although the vertebrae increase in size as the lumbar spine descends, none of the vertebrae themselves are specialized in any way like the aforementioned atlas and axis of the cervical spine. The L5 vertebra is not much different to the others other than in size, but since it is the most inferior vertebra in the spine, it takes more load bearing responsibility than any other vertebra in the spine making it a necessity to be the biggest and strongest [19,20].

The lumbar spine contains afferent and efferent nerves that are much more similar to those of the cervical spine, in that each one that comes out of the different levels have very specialized functions, which if damaged, can hinder an individual’s daily life and potentially leave them paralyzed from the waist down [21,22]. These nerves control mainly the front of the lower extremities, and when impinged can lead to loss of feeling, mobility, weakness, isolated lower back pain, and extending leg pain [23]. With all of the load bearing, torsion, and bending, these nerves tend to have the most significant chance to be impinged or damaged (roughly 95% in individuals aged 25–55 years, further discussed in Section 3.4), compared to any other spinal section [22,24]. 

### 1.4. Sacrum

The sacrum consists of five fused vertebrae (S1–S5) that connect to the pelvis at the sacro-iliac joint, and acts as the only skeletal connection between the trunk and the lower body [3]. While in adolescence, the sacrum remains unfused, as an individual grows into adulthood, the sacrum begins to fuse together. The fusion of the sacrum tends to begin with the lateral elements fusing around puberty, and the vertebral bodies fusing at about 17 or 18 years of age, becoming fully fused by 23 years of age [3], Figure 1. The sacrum has few active roles in the body, however one of those roles are incredibly vital, being the bridge between the hips with the rest of the spine [25].

Although the sacrum has no intervertebral discs, it does have very important afferent and efferent nerves that stem from the spinal cord, going through the entire lower extremity. The most important and commonly injured of these nerves travels through the L5/S1 space, which is more commonly known as the sciatic nerve. When this nerve is damaged or impinged it leads to pain and numbness down the legs hindering much of an individual’s way of life [26].

### 1.5. Coccyx

The coccyx consists of three to five fused vertebrae depending on the individual (four is most common) that are connected to the bottom of the sacrum, and is usually referred to as the tail bone [3], Figure 1. The coccyx’s major functions include acting as an attachment site for pelvic tendons, ligaments, and muscles, mainly those of which make up the pelvic floor, and supporting and stabilizing the body while in a sitting position [27].

The coccyx has no intervertebral discs nor do any nerves pass through it, therefore it is insignificant with regards to disc degeneration and disc damage. 

## 2. Intervertebral Discs

Every vertebra in the cervical (excluding the C1 and C2 vertebrae), thoracic, and lumbar spines is separated by intervertebral discs, each named for the two vertebrae they sit between (e.g., C6–C7, T7–T8, and L4–L5, also sometimes denoted as L4/L5). These discs make up about 20–30% of the total length of the spine, and have incredibly important functions including load cushioning, reducing stress caused by impact (shock absorber), weight dispersion, allowing for movement of individual vertebrae, and allowing for the passage of nutrients and fluid to the spine and spinal cord [28]. Although each disc grants almost identical functions to the spine, based on their location, their structure and mechanical properties change to adapt to the different loads, stresses, and strains produced [29]. For example, as the expected weight-bearing role of each disc increases, descending from the base of the skull along the length of the spine, the transverse cross-sectional area of the discs also increases. The pressure exerted on the discs however, does not increase to the same extent due to the fact that the cross-sectional area increases in the inferior direction [29].

Along with the changes in the cross-sectional areas of the discs, the height (thickness) of each disc changes throughout the spine as well. The cervical and lumbar spines have been shown to have much thicker discs than that of the thoracic spine, most likely being adapted to the higher range of motion expected from these sections, for both flexion-extension and torsion [29]. All cross-sectional areas and thicknesses for the continuation of this review will be associated with the transverse plane and disc height, respectively. 

On a smaller scale, the three components that form the disc, the annulus fibrosus, the nucleus pulposus, and the vertebral endplates, (further discussed in Section 2.2), change throughout the spinal sections as well [28]. For example, as the discs increase in thickness, the length of reinforcing fibers of the annulus fibrosus increase as well. This change allows for a decrease in fiber strain caused by a given movement for thicker discs compared to thinner discs [29]. Although there is a general trend between the structural and mechanical properties of the intervertebral discs and the spinal sections they belong to, each individual disc of the same section have their differences as well.

### 2.1. Classification of Intervertebral Discs

#### 2.1.1. Cervical Discs

The cervical spine consists of six intervertebral discs (C2/C3–C7/T1), with the absence of a disc between the atlas (C1) and the axis (C2) [3]. These discs are smaller in cross-sectional area than any of the other discs in the spine, due to the load bearing role of the cervical spine being much less than that in any other section, therefore decreasing the need for load distribution [29]. The average cross-sectional areas and thicknesses taken from 70 cervical discs range from 190–440 mm^2^ and 3.5 to 4.5 mm, respectively, shown in Table 1 [29,30]. 

In adults, the maximum flexion and extension of the cervical spine occurs around the C5/C6 disc, therefore its thickness is representative of such and will be, on average, thicker than the others. The cervical discs also show a maximum thickness in the anterior section and a minimum height in the posterior section, giving it a natural convex curvature [30]. Because of the mobility of the cervical spine, its discs have a significantly higher risk of damage from bending and torsion, making it the second most common spinal section for disc injury [34]. 

#### 2.1.2. Thoracic Discs

The thoracic spine consists of twelve intervertebral discs (T1/T2–T12/L1) [3]. These discs are greater in cross-sectional area than the cervical discs, however are still less than that of the lumbar discs. This is due to the amount of extra load transferred to the thoracic spine from the vertebrae above, therefore increasing the need for greater load distribution [29]. The average cross-sectional areas and thicknesses taken from 72 thoracic discs range from 500–1200 mm^2^ and 4.4 to 6.8 mm, respectively, shown in Table 1 [31,32]. 

Although the thoracic discs are greater in cross-sectional area than the cervical discs, they are still thinner in comparison. This is because the thoracic spine does not go through as much flexion/extension and rotation as the other sections of the spine, mainly due to the attachment of the rib cage [29]. The majority of the thoracic discs also show a greater height in the anterior section as opposed to the posterior section (exception of T4/T5, T5/T6, and T10/T11), like that of the cervical discs, however, the difference is not to the same extent as the other sections of the spine [31,32]. 

Because of the lack of mobility throughout the thoracic spine, its discs tend to have very little torsional stress, giving them a very low chance to become injured from degradation. However, if a high impact is sustained in the thoracic spine, there is a possibility of disc damage, although it is much more common for one of the vertebra to fracture before damage to the disc occurs [35].

#### 2.1.3. Lumbar Discs

The lumbar spine consists of five intervertebral discs (L1/L2–L5/S1) [3]. These discs have the greatest cross-sectional area out of all of the spinal sections, with L2/L3–L5/S1 being virtually equal. This is because the lumbar discs need to withstand the greatest amount of load without building up too much pressure and failing [29]. The average cross-sectional areas taken from roughly 1200 lumbar discs range from 1400–1700 mm^2^ and 7.6 to 9.4 mm, respectively, shown in Table 1 [32,33].

Like the cervical spine, the lumbar spine goes through a large amount of flexion/extension and torsion causing a high stress and strain on the discs. Due to these factors, they are the thickest discs and they have the largest surface area [32]. The lumbar discs also have a high ratio of anterior disc thickness to posterior disc thickness, the greatest being the L5/S1 disc, causing the lumbar spine’s natural convex curvature similar to the cervical spine [32,33]. Because of the mobility of the lumbar spine and the high loads applied to it, sometimes being in the order of thousands of newtons, its discs have a significantly higher chance of becoming damaged from bending and torsion, making it the most common spinal section for disc injury [36].

### 2.2. Intervertebral Disc Physiology

**Figure 2:**
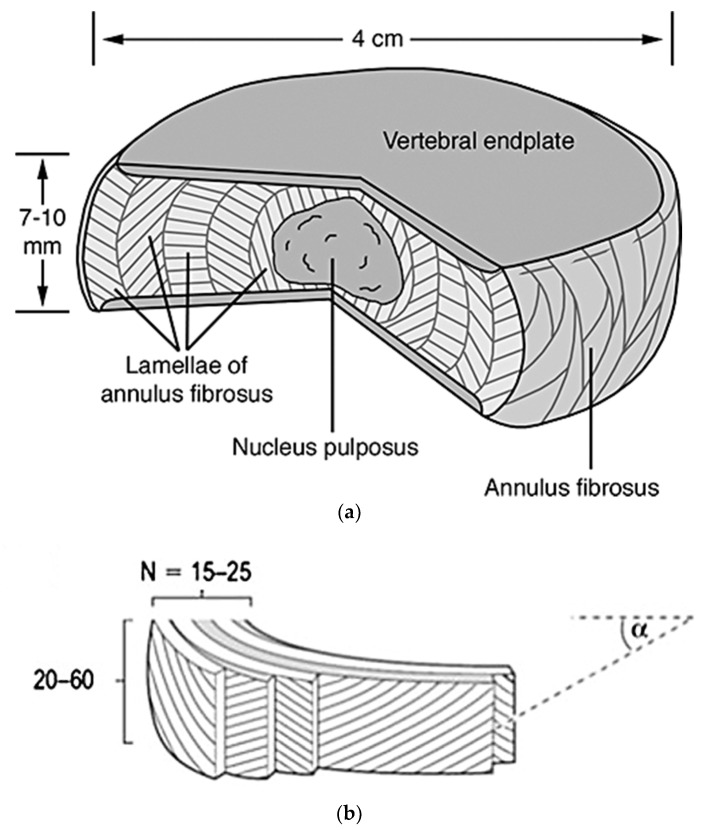
Pictured (**a**) is a cut out portion of a normal disc depicting the nucleus pulposus, vertebral endplates, and annulus fibrosus. The chosen intervertebral disc is 4 cm wide and 7–10 mm thick [37]. Depicted in the lower image (**b**) is a diagram showing the detailed structure of the annulus fibrosus, with its 15–25 lamellae comprised of 20–60 collagen fiber bundles. Also shown, is the angle α, which correlates to the directionality of the fibers’ bundles in relation to the vertebrae [38].

Each intervertebral disc is a complex structure comprised of three main components, a thick outer ring of fibrous cartilage called the annulus fibrosus, a more gelatinous core called the nucleus pulposus, and the cartilage vertebral endplates. All together, they bring structural and mechanical integrity to the organ. These components combine to give the necessary structural and mechanical properties to the intervertebral discs as a whole (further discussed in Section 2.2.1, Section 2.2.2 and Section 2.2.3) [37], Figure 2.

The intervertebral discs are the among the largest avascular tissues within the body, due to the lack of vessel penetration throughout the internal sections. Therefore, a flow of nutrients occurs via diffusion from the pre-disc vessels that reach into the outer most layers of the disc [37]. The increase in vascularization into the inner parts of the discs are contributed to their degeneration (further discussed in Section 2.2.4 and Section 3). To better understand the functions and properties of each component, they will be further described in detail. 

#### 2.2.1. Annulus Fibrosus

The annulus fibrosus is a fibrocartilaginous tissue that is structured as concentric rings, or lamellae, surrounding the nucleus pulposus (Figure 2), and is referred to as having two main sections, the inner and outer annulus fibrosus. Both of these sections are composed of mostly water (70–78% inner and 55–65% outer wet weight), collagens (type I and type II collagen, 25–40% inner and 60–70% outer dry weight), proteoglycans (11–20% inner and 5–8% outer dry weight), and other minor proteins building-up the extracellular matrix (ECM). The composition of the ECM varies gradually with increasing radial distance from the nucleus, mainly the type of collagen (having more collagen type I as the distance increases) and decrease of proteoglycans [37,39,40]. These ECM components help create the more rigid structure of the annulus fibrosus necessary to withstand the loads and strains applied. 

The annulus fibrosus accounts for a multi-layered structure with alternating collagen fiber angles (varying in degrees throughout the lamella) that help creating a structurally stable material, housing the nucleus pulposus, keeping it under pressure and from impinging on the spine, and enabling the disc to withstand complex loads with its inhomogeneous, anisotropic, and nonlinear mechanical behaviors [41].

##### (1) Composition

The annulus fibrosus is a unicellular tissue comprising of annulus fibrosus cells embedded in an ECM composed mainly of collagen types I and II, and proteoglycans, which are responsible for the high load-bearing properties of the tissue [41]. Collagens play structural roles, contributing to the mechanical properties, tissue organization, and shape of the annulus fibrosus. Many different isoforms of collagen exist, more than 28 of which have already been identified. It is one of the most abundant ECM proteins in the body, and can take varying structures such as fibrils, short-helix or globular structures [42]. The annulus fibrosus contains only fibril forming collagen, collagen type I and type II, which form the fibrocartilage of the lamellae, Table 2. The collagen types I and II replace one another in a smooth gradient, transitioning from 100% type I in the furthest outer lamella, to 100% type II in the furthest inner lamella [40]. However, based on discs of different individuals, some might include minute amounts of the opposing collagen in the inner and outer lamellae. Not only does the type of collagen change as radial distance increases, but the concentration of collagen as well, increasing from inner annulus to outer annulus [40]. This creates a smooth transition zone between the softer nucleus pulposus and the stronger outer annulus fibrosus [43].

All collagen consists of a triple helix structure comprised of three polypeptide chains [45]. These polypeptide chains, called alpha (α) chains (procollagens), further diversify the collagen family by creating several molecular isoforms for the same collagen, as well as hybrid isoforms comprised of two different collagen types. The size of these α chains can vary from 662 to 3152 amino acids for humans, and can either be identical to form homotrimers or different to form heterotrimers [42]. Collagen type I is considered a heterotrimer consisting of α1(I) and α2(I), while collagen type II is considered a homotrimer consisting of only α1(II), both of which are found in the annulus fibrosus. 

**Figure 3:**
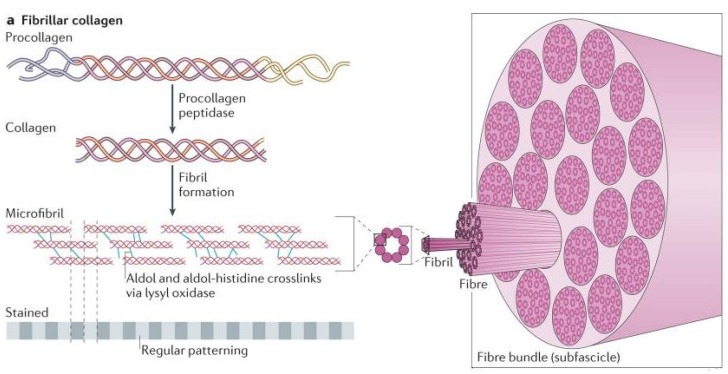
Construction of fibrillary collagen as described above [45].

After the transcription and translation of the procollagen α chains, four distinct stages occur for the assembly of collagen fibrils. The first stage is transportation of the α chains into the rough endoplasmic reticulum, where they are modified to form the triple-helical procollagen. The second stage is the modification of the procollagen in the Golgi apparatus and its packaging into secretory vesicles. The third stage is the formation of the collagen molecule in the extracellular space by cleavage of the procollagen. The final stage is the crosslinking between the collagen molecules to stabilize the supramolecular collagen structure [45], Figure 3. These collagen fibrils are vital to the structure, strength, and flexibility of the fibrocartilage in the annulus fibrosus lamellae. 

Proteoglycans are glycosylated proteins which have covalently attached highly anionic glycosaminoglycans (GAGs). Major GAGs include heparin sulphate, chondroitin sulphate, dermatan sulphate, hyaluronan, and keratin sulphate [45]. They are less abundant glycoproteins found in the annulus fibrosus ECM, and instead of being predominantly fibrillar in structure, like collagen, they form higher ordered brush-like ECM structures around cells. The main proteoglycans present in the annulus fibrosus are aggrecan and versican, which promote hydration and mechanical strength within the tissue. The keratin sulphate and chondroitin sulphate attached to their protein cores provide the ability to aggregate to hyaluronic acid, resulting in substantial osmotic swelling pressure crucial for the biomechanical properties of the tissue [45,46]. To clarify, their major biological function is to bind water to provide hydration and swelling pressure to the tissue, giving it compressive resistance. More specifically, the negative charges of the sulfated and carboxylated GAGs help trap water within the brushes, generating large drag forces when a load is applied to the tissue, as well as creating osmotic pressure for added resistance [46]. Inverse of the collagen, the proteoglycan concentration has an increasing gradient from outer annulus to inner annulus, or transition zone [40]. Other proteoglycans present in smaller amount on the ECM are the small leucine-rich repeat proteoglycans (SLRPs), such as decorin and byglycan, which are implicated in fibrillary collagen assembly. 

##### (2) Structure

**Figure 4:**
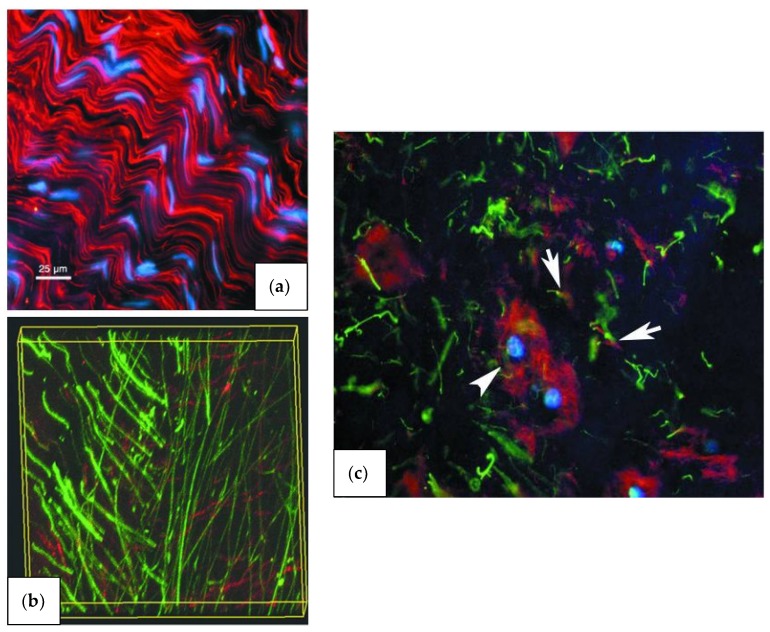
Fluorescence microscopic images of stained components in the outer annulus fibrosus (**a**), inner annulus fibrosus (**b**), and nucleus pulposus (**c**). The microfibrils in relation to cell distribution (blue) and collagen fiber organization (red) indicates the organization of the microfibrils within the ECM of the outer annulus fibrosus. Opposite however, the microfibrils (red) and elastin fibers (green) in the inner annulus fibrosus do not demonstrate any organization or co-localization to any great degree within the ECM. These two distinct characteristics of organization give rise to the varying mechanical properties of each, Section 2.2.1, (3). The microfibrils (red) show a tendency to hover/organize around the nucleus pulposus cells (blue), while the elastin fibers (green) have a tendency to stay dispersed through the entire ECM [51].

The annulus fibrosus has a unique structure consisting of anywhere from 15 to 25 distinct layers (lamellae), depending on the circumferential location, the spine level, and the individual’s age, with the thickness of these individual lamellae varying both circumferentially and radially, increasing as age increases [47]. Each adjacent lamella is held together by discrete collagenous bridging structures comprised of type VI collagen, and aggrecan and versican, which are orientated radially to wrap around individual collagen fibers and prevent severe delamination [48,49]. Based on the location of the disc, the amount of collagen fibril bundles in each lamella can vary from 20 to 60 bundles over the total height of the disc, with an average inter-bundle spacing of 0.22 mm and bundle thickness of roughly 10 microns [47], Figure 2. These bundles sit at different angles ranging anywhere from 55° to 20°, alternating direction every other layer, and have a planar zig-zag (crimped) structure. This allows them to be stretched and extend more as the crimps straighten out, resulting in the rotational and flexion/extension mobility of the spine [48,50]. Although the components within the annulus fibrosus are relatively the same, as previously stated, the organization of components such as microfibrils, collagen fibers, and elastin fibers differ with respect to the outer and inner annulus fibrosus [51]. This gives rise to different mechanical properties throughout the structure, detailed in Table 3 and Figure 4 [50,52,53].

The annulus fibrosus’ unique structure helps give it its mechanical functions of containing the radial bulge of the nucleus, enabling a uniform distribution and transfer of compressive loads between vertebral bodies, and to distend and rotate, allowing and facilitating joint mobility [40].

##### (3) Mechanical Properties

Like the collagen and proteoglycan concentration, the mechanical properties of the annulus fibrosus differ with an increase in radial distance, usually becoming stronger and stiffer towards the outer annulus. These mechanical properties are highly anisotropic and nonlinear in uniaxial tension, compression, and shear, and have a high tensile modulus in the circumferential direction [52]. In particular, the tensile properties of the lamella show drastic differences depending on the tested samples and the orientation at which they are tested. When testing parallel to the alignment of the collagen fiber bundles as opposed to perpendicular, the strength and modulus increases due to the strength and reinforcement given by the fibers, and the same correlation can be found when testing the outer lamellae as opposed to the inner lamellae, Table 3 [50,52,53,54,55].

Although the elastic modulus of the lamella differs by a factor of roughly 500, with respect to fiber orientation, when tested as a whole, the tensile elastic modulus instead hovers around 18–45 MPa [52,53]. As the stress induced on the annulus fibrosus increases, the rigidity of the system increases. This mechanical behavior is the result of the un-crimping of the collagen fibers that leads to the stiffening of the intervertebral disc tissue for larger strains. Not only does the stiffness relate to amount of strain on the annulus fibrosus, but also the load rate of the induced stress [54].

The annulus fibrosus is the only section of the disc that undergoes tensile stress, and it is usually due to these stresses that the collagen fibrils breakdown and deteriorate, making its unique tensile properties a focus when studying disc degeneration. However, while tensile properties are important for the understanding of how much stress and strain the annulus fibrosus can withstand, the injuries sustained are rarely due to a single impact, but more often the cyclic loading or wear and tear of the spine that causes deterioration of the collagen fibrils [50,53]. Therefore, cyclic loading tests are crucial for the understanding of the annulus fibrosus’ mechanical integrity and resiliency of the tissue. For example, both the anterior and posterior sections of a healthy annulus fibrosus have been shown to withstand more than 10,000 applied cycles with a stress magnitude of 45% or less of its ultimate tensile strength [50].

Although not as important for the annulus fibrosus as it is for the nucleus pulposus, compressive stresses and strains still occur on the lamellae, Table 3. However, they have very little effect on the degradation of the annulus fibrosus. Most often only the swell pressure (P_sw_), modulus (H_A_), and permeability (k) are characterized [55].

#### 2.2.2. Nucleus Pulposus

The nucleus pulposus resides in the middle of the disc surrounded by the annulus fibrosus, which keeps it from leaking into the spinal canal. It consists of randomly organized collagen type II fibers (15–20% dry weight) and radially arranged elastin fibers, housed in a proteoglycan hydrogel (50% dry weight), with chondrocyte-like cells interspersed at a low density of approximately 5000/mm^3^ [37,56]. The nucleus is an incompressible structure that it is made up of about 80–90% water, which helps it carry out its vital roles in the intervertebral disc of compressive load dispersion, compressive shock absorption, and keeping the inside of the disc swollen for necessary internal pressure [57]. 

##### (1) Composition

There are four main components found in the nucleus pulposus; collagen type II fibrils and elastin fibers (roughly 150 micrometers in length), proteoglycans, and chondrocyte-like cells. Each play a vital role in the performance and health of the nucleus pulposus, providing it with the necessary mechanical properties to serve its functions [58]. For a description of collagen and proteoglycan formation and structure, the reader is referred to Section 2.2.1, (1).

Unlike the annulus fibrosus, the collagen in the nucleus forms a loose network, which is joined by the network of elastin fibers. The elastin fibers are necessary for maintaining collagen organization and recovery of the disc size and shape after the disc deforms under various loads. It accomplishes this with its unique structure of microfibrils forming a meshwork around a central elastin core, Figure 4. These microfibrils are structural elements of the nucleus’ ECM, and have been found distributed in connective and elastic tissues such as blood vessels, ligament, and lung [51]. 

The microfibrils play vital roles in the properties of the elastic fibers, such as conferring mechanical stability and limited elasticity to tissues, contributing to growth factor regulation, and playing a role in tissue development and homeostasis. Microfibrils are made up of a multicomponent system, consisting of a glycoprotein fibrillin core (three known types), microfibril associated proteins (MFAPs), and microfibril associated glycoproteins (MAGPs). The MFAPs and MAGPs, as well as a few other peripheral molecules, contribute to link microfibrils to elastin, to other ECM components, and to cells [59].

In the nucleus pulposus, the chondrocyte-like cells act as metabolically active cells that synthesize and turnover a large volume of ECM components, mainly collagen and proteoglycans [60]. They produce and maintain the ECM with the presence of Golgi cisternae and well-developed endoplasmic reticulum, and are able to withstand very high compressive loads and help with the movement of water and ions within the matrix [61]. They also maintain tissue homeostasis, play a role in the physio-chemical properties of cartilage-specific macromolecules, and prevent degenerative diseases like degenerative disc disease and osteoarthritis. However, with age these cells start to become necrotic, increasing from about 2% at birth to 50% in most adults. This can lead to cartilage/collagen degradation, abnormal bone growth formation on the vertebrae (osteophyte) where bone on bone friction occurs, and stiffening of joints [58,60]. 

##### (2) Structure

The nucleus pulposus is a soft, gelatinous mass that is irregularly ovoid and is found under pressure in the center of the disc. Because it is mostly water (between 80–90%), it does not have a definite structure or form, but like a liquid, takes the shape of wherever it is confined [62]. From birth to adolescence, the nucleus pulposus is a semi-fluid mucoid mass formed by proliferation and degeneration of embryological notochord cells with a few scattered chondrocytes and collagen fibers. As age increases into adulthood, the notochord cells completely degenerate and become replaced by chondrocyte-like cells, which deposit a specialized ECM to provide the nucleus tissue with its structure and mechanical properties. Also with age, the nucleus becomes less fluid-like and more cartilaginous as the collagen fibrils start to crosslink together forming fibers like the collagen type II fibers of the annulus [63]. 

##### (3) Mechanical Properties

Being a virtually incompressible liquid, the nucleus pulposus does not endure any tensile stresses or strains, and the loads it can withstand in compression are largely due to the force that the annulus fibrosus can resist radially. The natural swell pressure of the nucleus at rest is 0.138 MPa, which is correlated to the water uptake and retention during resting periods. However, as compressive forces are introduced to the nucleus, the swell pressure increases to withstand the loads within the confined space of the annulus fibrosus [52]. When testing for compressive properties, the nucleus is confined so that accurate measurements can be taken, Table 3. Confining the nucleus during testing allows for a more accurate resemblance of the resistance towards outward deformation controlled by the annulus fibrosus, as well as keeping the nucleus from being infinitely compressed, since it is a virtually incompressible liquid.

During everyday activities, the lumbar compressive forces can fluctuate between 800 N and 3000 N. This causes the nucleus to become pressurized up to 0.4 MPa while lying down, 1.5 MPa while standing or sitting, and up to 2.3 MPa while actively lifting, however these stresses can vary slightly due to the different dimensional areas of the disc [64]. Although the mechanical testing of the nucleus pulposus is not quite as extensive as that of the annulus fibrosus, it does not make it any less important to the structural and mechanical properties of the disc as a whole.

#### 2.2.3. Vertebral Endplates

**Figure 5:**
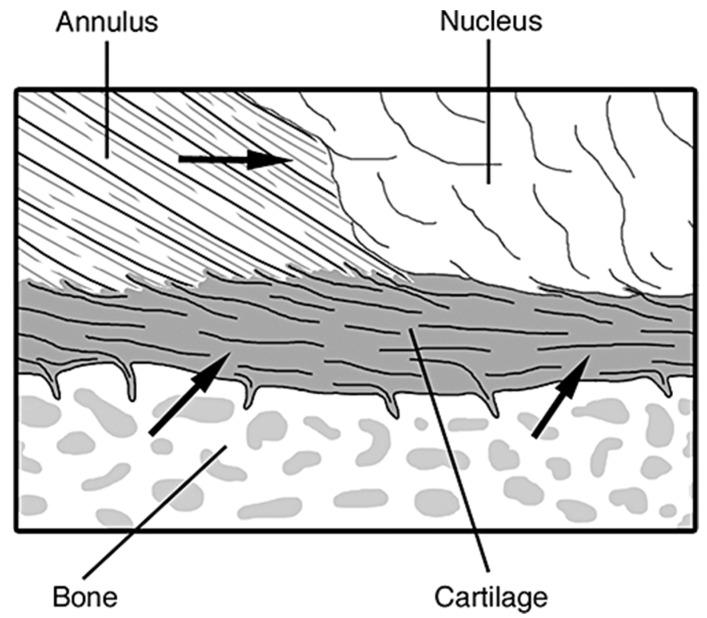
The connection of the hyaline cartilage vertebral endplate to the perforated cortical bone of the vertebral body and collagen fibers of the annulus and nucleus. The arrows in the figure refer to the direction of nutrients and blood flow through the different components of the disc, mainly coming from the bone through the vertebral endplates [37].

The vertebral endplates are situated on the top and bottom of each intervertebral disc, and are comprised of hyaline cartilage [65]. Their main function is to function as an interface between the dense, harder cortical bone shell of the vertebrae and the annulus and nucleus via mechanical interlocking, and to keep the nucleus pressurized and from bulging into the soft, spongy/cancellous trabecular bone center of the vertebrae, Figure 5. The vertebral endplates are the strongest part of the intervertebral disc, and usually fail after the vertebral body has already fractured [38]. 

The vertebral endplates also have the unique role of acting as the main transport for nutrients in and out of the disc. This provides the nucleus and annulus with the cells and other required components that keep the disc alive, and from degenerating [64]. 

##### (1) Composition

The vertebral endplates are composed of an osseous and a cartilaginous component. The hyaline cartilage within differs from the articular cartilage of the joins on its structure. While both are composed of chondrocytes, proteoglycans and a string collagenous network, the former is not connected to the underlying bone [65]. The hyaline cartilage of the vertebral endplates maintains very similar macromolecules in their ECM as that of the nucleus pulposus, however the ratios of proteoglycan to collagen content differs drastically. The typical ratio of glycosaminoglycan to collagen in the endplates is roughly 2:1, providing to the tissue with higher mechanical properties than the nucleus pulposus with a ratio of 27:1 [66]. Also, distinctively different from the annulus fibrosus’ fibrocartilage which contain large collagen fiber bundles, the endplates have fine collagen fibers similar to the nucleus, but they are closely packed together. The hyaline cartilage in the endplates are made up of multiple types of collagen. Collagen Type II is the main collagenous component on the endplates. Collagens are often employed as a measure of the degeneration state (hypertrophy of chondrocytes and ossification) of the endplate, being the downregulation of collagen II and upregulation of collagen X the most characteristic markers. [65]. The other collagens, Type I, III, V, VI, IX, and XI are present in small amounts, and only contribute to a minor portion of the cartilage with the main functions of forming and stabilizing the collagen Type II fibril network [67,68,69].

All of the collagen structures and cellular make-up are the same for the hyaline cartilage as previously discussed in the annulus fibrosus (Section 2.2.1, (1)).

##### (2) Structure

Two major structures can be distinguished in the vertebral endplates, the collagen fibers of the cartilaginous section (roughly 0.1 to 0.2 mm thick) that connect to the annulus fibrosus and the bony layer of the vertebral section (roughly 0.2 to 0.8 mm thick) that connect to the vertebrae. For the cartilaginous section, the proteoglycan hydrogel-enveloped collagen fibers run horizontal and parallel to the vertebral bodies, however the fibers then continue into the annulus fibrosus at an angle parallel to the currently residing fibers [37]. The integration between the collagen fibers in the nucleus and the endplates is more convoluted. For the vertebral section, the bony component of the endplate is a porous layer of fused trabecular bone with osteocytes embedded within saucer-shaped lamellar packets, resembling the structure of the vertebral cortex [64]. 

The most important structural features of the endplate biomechanical functions are the thickness, porosity, and curvature. For example, thick, dense endplates with a high degree of curvature are stronger than thin, porous, and flat endplates [64]. They are typically less than 1.0 mm thick, and cover the entire surface area of the top and bottom of the intervertebral disc. The thickness across the width of the disc is not uniform, varying considerably, while tending to be the thinnest in the central region adjacent to the nucleus [65]. The density tends to increase towards the vertebral periphery where the subchondral bone growth starts, however porosity can increase up to 50–130% with aging and disc degeneration. Due to the variations throughout the structure of the vertebral endplate, its mechanical properties vary as well [70,71].

##### (3) Mechanical Properties

The mechanical properties of the vertebral endplates vary with the region on which the endplate is tested, as well as the region of the spine from which they are extracted. The central area of the endplates tends to be the weakest, and increases in strength and stiffness radially towards the outer annulus [70,71]. When tested in different sections of the spine, the endplates show a significant increase in strength and stiffness from superior to inferior sections of the spine. Not only do the properties change between spinal sections, but also within the same section, such as the stiffness and strength increasing as the lumbar spine descends (L1/L2–L5/S1) [70]. Due to the unique structure of the endplates, they are able to withstand high loads, outlasting the vertebral body more often than not. The failure of the vertebral endplate tends to occur at around 10.2 kN, however the failure of the vertebral body, usually due to fracture, occurs around 4.2 kN in individuals 60 years of age or older, and around 7.6 kN in individuals 40 years or younger [47,72]. Not only do the endplates have great strength, but they also possess great stiffness (1965 ± 804 N/mm) that allow it to be semi-flexible during the loads put onto the spine. This helps the nucleus move and cushion loads more readily inside of the disc, while also protecting the endplates from tensile damage, of which they are most likely to fail [64,73]. 

#### 2.2.4. Blood Vessels and Nerve Supply

**Figure 6:**
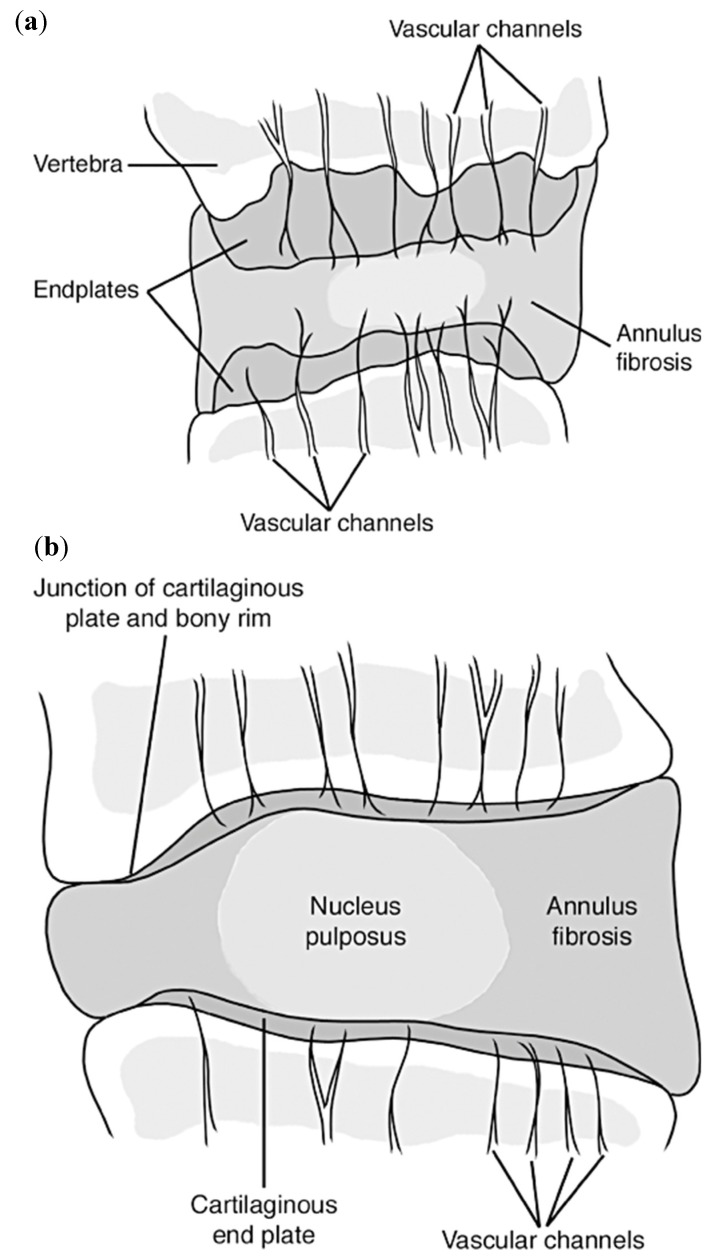
(**a**) Schematic representation of the multiple longer and thicker vascular channels throughout the intervertebral disc on a 10-month old female; while (**b**) represents the vascular channels throughout the disc of a 50-year old adult, showing the retraction and thinning of the channels [37].

Because the intervertebral disc is one of the most avascular tissues in the human body, in a healthy adult, it tends to have very few microvessels. However, during early stages of skeletal development, blood and lymph vessels are present throughout the majority of the disc with the exception of the nucleus. With maturation of the skeleton, blood and lymph vessels found within the disc start to decrease and migrate towards the outer parts of the annulus fibrosus. These blood vessels extend through the cartilaginous endplates into the inner and outer annulus and slightly into the nucleus up to 12 months of age. However, as age increases past 12 months into skeletal maturity (around 20 years of age), the blood vessels start to recede from the nucleus and inner annulus, until they only remain in the outer annulus and endplates, Figure 6 [37,74]. 

Given the size of the tissue, once the blood vessels retract from the disc in adulthood, the discs rely on diffusion through the endplates and annulus for the nutritional supply of the disc cells [75]. This reduced nutrient supply is thought to contribute to the degeneration of the discs and to be responsible of the lower regenerative potential of the tissue during aging, giving a reason for the low structural and functional restoration properties of the tissue during aging [75]. 

**Figure 7:**
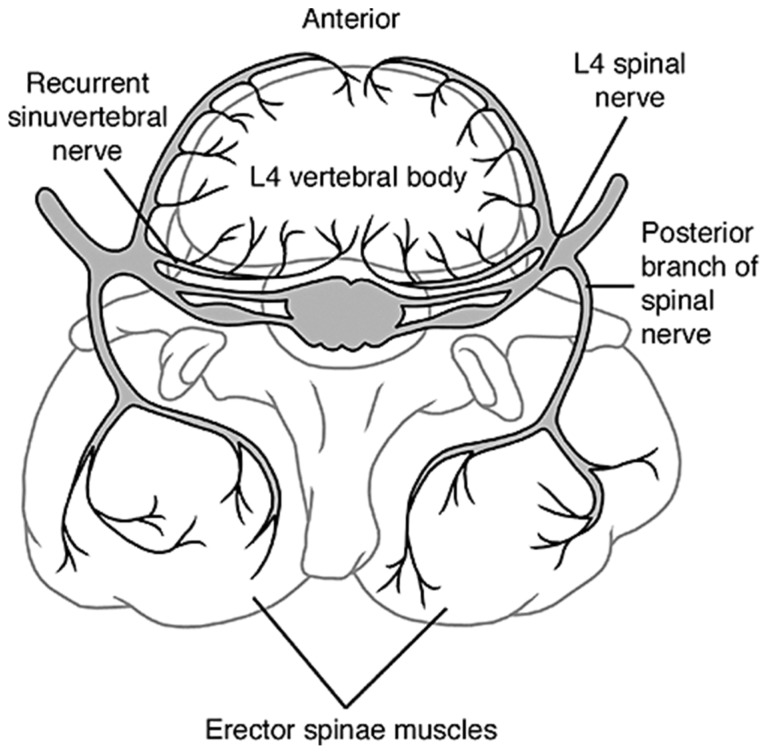
The innervation of a healthy intervertebral disc, showing the sinuvertebral nerves and rami communicantes extending into the vertebral foramen and the outer annulus of the disc [37].

The intervertebral discs are innervated organs with some of the most important nerves residing in the cervical and lumbar spine. Recurrent sinuvertebral nerves innervate the posterior and some of the posterolateral aspects of the disc, and the posterior longitudinal ligament, branching off the dorsal root ganglion extending from the spinal cord. The other posterolateral aspects receive branches from the adjacent ventral primary rami and from the grey rami communicants [76]. Lateral aspects of the disc receive other branches from the rami communicantes, some of which cross the intervertebral disc and are embedded within the surrounding connective tissue of the disc, such as the origin of the psoas for the lumbar spine. Lastly, the anterior aspects along with the anterior longitudinal ligament are innervated by recurrent branches of rami communicantes, Figure 7 [76]. 

Opposite of blood vessels, in a healthy young adult, the sensory nerve endings of the disc can be found on the superficial layers of the annulus and in the outer third of the annulus, only extending about 3 mm into the disc [77]. With age and degeneration, the nerves tend to creep into the inner parts of the disc by means of neoinnervation, arising from granulation tissue growing in the disc. This can cause innervation of the middle and inner annulus, and potentially of the nucleus pulposus. As innervation progresses, significant problems with regards to lower back pain can arise from the amount of pressure being induced onto the discs, and therefore pressure onto the nerves [77,78].

## 3. Spinal Degeneration and Lower Back Pain

Back pain is a major health problem in Western industrialized societies, inflicting suffering and distress on a large number of patients, especially those of old age, increasing with the increased aged population. The effects of this problem are vast, with a study in the year 2000 in the UK showing prevalence rates ranging from 12% to 35%, and around 10% of sufferers becoming chronically disabled [79]. With total costs, including direct medical costs, insurance, lost production, and disability benefits, reaching into the billions of dollars, an enormous economic burden is placed on society [79]. In the United States alone, costs associated with lower back pain exceeds $100 billion per year, two-thirds resulting from lost wages and reduced productivity [80]. Among the other third are direct costs for medical treatments of back pain diagnoses, estimated at $34 billion out of the total $47 billion for all treatments for pain diagnoses in 2010. These costs include office-based visits, hospital outpatients, emergency services, hospital inpatients, and prescription drugs [81]. This back pain is strongly associated with disc degeneration and injury, the majority of the time occurring in the lumbar spine due to the increased stresses, strains, and torsion compared to other sections, and the thoracic spine being the least affected [11]. 

Intervertebral discs can degenerate due to injury or due wear and tear, as a result of the stress and strain to which the tissue is exposed to on a daily basis. However, intervertebral discs are among the most avascular tissues in the human body and together with the low proliferative potential of cells within, being almost quiescent, results in a tissue that is unable to adequately self-regenerate [82]. Multiple factors promote the degeneration of the tissue other than just wear and tear, such as genetic predisposition, impaired metabolite transport, altered levels of enzyme activity, cell senescence and death, changes in matrix macromolecules and water content, osteoarthritis, structural failure, and neurovascular ingrowth. Although genetic inheritance is the greatest risk factor, it does not cause discs to degenerate by itself, but instead increases their susceptibility to environmental factors such as high and repetitive mechanical loading and smoking cigarettes [83]. 

### 3.1. Degenerative Disc Disease

Degenerative disc disease is defined by the degeneration of intervertebral discs due to aging and other environmental factors, with genetic inheritance playing a significant role in the rate of degradation. Approximately 50–70% of the variability in disc degeneration is caused by an individual’s genetic inheritance [83,84]. The inherited genes associated with disc degeneration include those for collagen type I and IX (COL1A1, and COL9A2 and COL9A3, respectively), aggrecan, vitamin D receptor, matrix mettalopeptidase-3 (MMP3), and cartilage intermediate layer protein (CILP). The strength of musculoskeletal tissue, like that of intervertebral discs, is affected by the composition of the ECM, such as the strength of the collagen fibrils throughout the annulus fibrosus, which is regulated by the aforementioned genes (and others) [85]. Although an unfavorable genetic inheritance is present at birth, disc degeneration only becomes prevalent and common in the individual’s 40’s, and usually only in the lower lumbar spine [83,84]. Some individuals however, can become inflicted by this disease much earlier than the norm, depending on both the severity of their genetic deficiencies and lifestyles. 

Degeneration of intervertebral discs can occur at faster rates than for other tissues and is sometimes presented on individuals as young as 11–16 years of age, usually found in the lumbar section [79]. Degenerative disc disease affects about 20% of people in their teens, showing mild signs of degeneration before their second decade of life. However, because the discs have yet to undergo progressive innervation, most cannot feel the pain and disabilities associated with degeneration until it propagates through to the later years of life. Therefore, this disease increases drastically with age, causing the discs of around 10% of 50-year-old population and 60% of 70-year-old population to become severely degenerated, significantly hindering daily activities [79]. 

**Figure 8:**
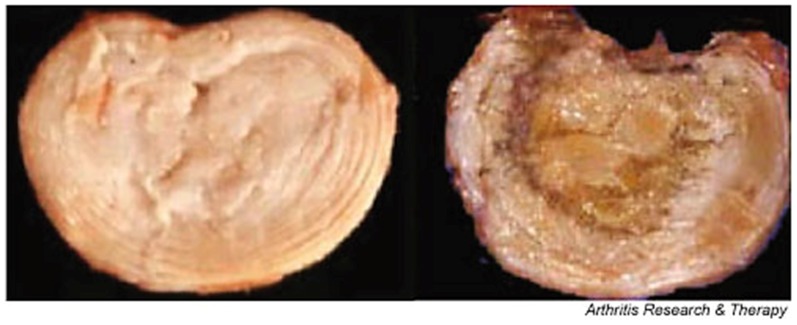
A healthy, normal intervertebral disc on the left, shows a distinct difference between the swollen, softer looking nucleus and the ringed annulus. However, during growth and skeletal maturation, the boundary between these components becomes less obvious, and with the nucleus generally becoming more fibrotic and less gel-like, like the highly degenerate disc on the right [79].

Degenerative disc disease can affect the tissue in many ways, causing it to undergo striking alterations in volume, shape, structure, and composition that result on a decreased motion and an altered biomechanical properties of the nucleus pulposus and annulus fibrosus tissues, thus altering the mechanics of the spine [84]. Both the nucleus pulposus and annulus fibrosus experience changes individually, mainly in the ECM composition and structure. Consequentially, due to the compositional changes on the discs ECM, such as collagen, proteoglycan, and water content, the major structural properties become hindered as well. The main structural effects tend to be the loss of swelling ability, and therefore volume of the nucleus, and tears or fissures forming in the annulus [86]. When these fissures are formed in the annulus, there is also frequently a cleft formation of some sort, particularly in the nucleus, and the morphology becomes more and more disorganized, Figure 8. The vertebral endplates also go through some deformation and changes, such as an increase of porosity from 50 to 130%, the natural curvature becoming less apparent and flattening out, and a significant decrease in the thickness by roughly 20 to 50% [64,71]. These changes make the vertebral endplate much more likely to fracture under the stresses of the spine and tensile stresses induced by the nucleus. 

Along with major structural changes, many biochemical changes occur throughout the disc as well. With age and degeneration, comes an increased incidence in these changes, including cell proliferation and death, mucous degeneration, decrease in proteoglycan content, increase in collagen fibril cross-linking (mainly nucleus), granular changes, and concentric tears in the annulus [79]. Innervation and vascularization of the disc are thought to cause the increase in cell proliferation in the nucleus, which leads to the formation of clusters of living, necrotic, and apoptotic cells. The appearance of these apoptotic and necrotic cells can promote cell death in the healthy living cells. Unfortunately, these mechanisms tend to be very common with age, with more than 50% of cells in adult discs being necrotic [79].

As degeneration progresses, compositional and structural changes to the discs become more and more apparent. The status of the degeneration is commonly studied via Magnetic Resonance Imaging (MRI) and evaluated with the Magnetic Resonance Classification System with rankings from Grade I to Grade V [87]. The ranks are based on disc structure, signal intensity, distinction between the nucleus and annulus, and the height of the disc, Table 4.

**Figure 9:**
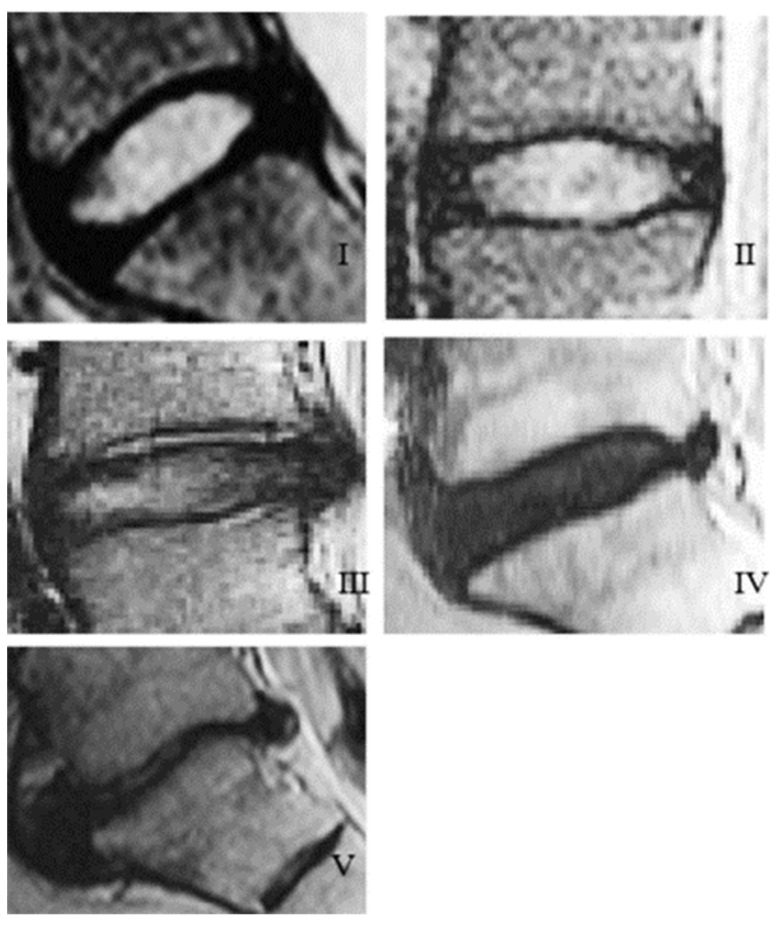
MRI scans showing the different grades of disc degeneration based on the Pfirrmann grading system, (**I**–**V**) referring to Grades (**I**–**V**): I is representative of Grade (**I**) degeneration, (**II**) is representative of Grade (**II**) degeneration, (**III**) is representative of Grade (**III**) degeneration, (**IV**) is representative of Grade (**IV**) degeneration, and (**V**) is representative of Grade (**V**) degeneration [88].

Although the grading scale has shifted from the previous radiographic imaging systems, which focuses on the antero-posterior abnormalities of the discs, distinguishing among bulging, protrusion, and extrusion, (Grade I through Grade III respectively), the MRI images used for the Magnetic Resonance Classification System still show the symptoms of all three past grades, Figure 9. It can be seen that Grade II–III shows a slight bulging of the nucleus (more prominent in Grade III), Grade IV shows the beginning stages of protrusion of the disc, and Grade V shows a fully blown-out disc in which the entire nucleus has been extruded into the spinal canal [87]. 

### 3.2. Osteoarthritis

Although not as common of a cause for disc degeneration as degenerative disc disease, osteoarthritis can have a significant impact on the structural changes of the intervertebral discs, causing major problems at long term. Osteoarthritis is a degenerative disorder of the articular cartilage affecting over 30% of the population above the age of 65 and is associated with hypertrophic changes of the tissue affecting the facet joints and vertebrae of the spine, especially the lumbar spine [89,90]. Many risk factors can affect the probability as well as severity of osteoarthritis including genetic inheritance, female gender, past physical trauma, increased age, and obesity. Symptoms usually include joint pain that increases with movement, trouble or disability with activities of daily living, and lower back pain associated with narrowing disc space. With the current U.S. population living longer and becoming more obese, osteoarthritis has become more common than it ever has before, affecting an estimated 27 million adults in the U.S. [89,91]. 

Peripheral joints such as hips, knees, and hands, were most commonly thought of with regards to osteoarthritis, with prevalence in the spine often being ignored. However, the prevalence of disabilities and functional distress caused to the spine by osteoarthritis are actually quite high. In the lumbar spine, it is a very common condition, with a prevalence range of roughly 40–85% based on age, weight, and other factors. The spinal degeneration process has been partly linked to both osteoarthritis and changes in facet joint structure. Osteoarthritis leads to the narrowing of disc spacing from the formation of vertebral osteophytes introducing increased pressure to the disc. Being comprised of the same type of cartilage as appendicular joints, facet joints have similar pathological degenerative processes, such as crystal deposition within the cartilage, degradation from high impact and torsional loads, and joint instability, which all can cause additional stress to the discs [91]. Both the intervertebral discs and facet joints play vital roles in the motion of the spine, especially in the cervical and lumbar spines, therefore when they are heavily affected by osteoarthritis, the mobility of the spine can decrease significantly, and pain can ensue from even the slightest of movements. 

Three main components are observed with regards to osteoarthritis in spine, referred to as the “three joint complex”. These components include the structure of vertebral osteophytes, facet joint osteoarthritis, and disc space narrowing. With the amount of nerve supply running through all of these spinal structures, lower back pain can be generated by any of them [91]. With further progression of disc degeneration in the spine, the facet joints as well as vertebrae further degenerate, due to disc space narrowing, which in turn puts even more stresses onto the intervertebral discs. Facet joint osteoarthritis is a multifactorial process that is highly affected by disc degeneration, leading to greater loads and motions endured by the joints [92]. This, consequently, leads to the breakdown of the layer of hyaline cartilage between the two subchondral bones, creating friction and grinding between them, and finally abnormal bone growth and pressure. However, facet joint osteoarthritis can still occur in the absence of disc degeneration, in which case it causes more stress and motion on the intervertebral disc leading to quicker degeneration [93]. 

**Figure 10:**
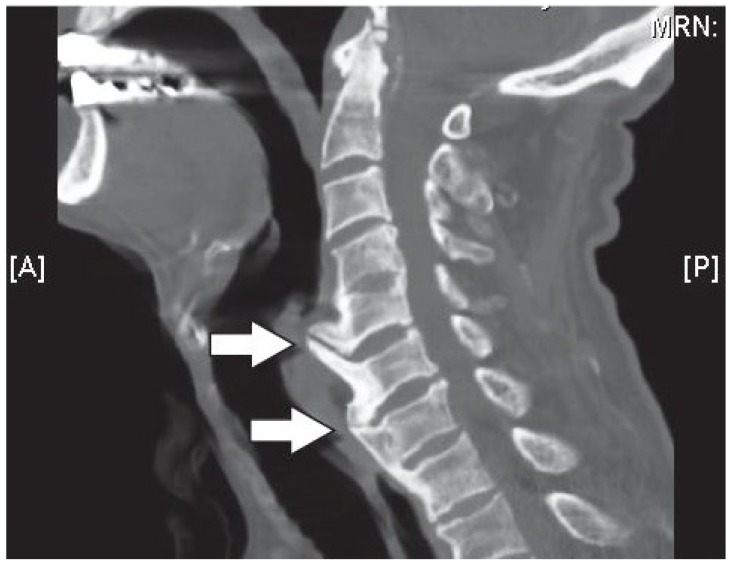
Sagittal computerized axial tomography (CT scan) image of the cervical spine showing large anterior osteophytes (indicted by the arrows) extending from C5 to C7, which affect the intervertebral disc space [95].

Changes in the structure of the vertebral osteophytes on the shape of formation of bony outgrowths which arise from the periosteum at the junction of the bone and cartilage, lead to disc space narrowing, Figure 10. Although it is highly correlated to disc degeneration, like that of the osteoarthritis in the facet joints, osteophyte formation in the vertebral column can occur without any signs of cartilage damage, implying that with the general aging process, they may form in an otherwise healthy joint [91]. In this case, the vertebral osteophytes can cause extra stresses on the discs, mainly in the annulus fibrosus, potentially weakening it for further degeneration, damage, and tears/fissures [91,94]. 

Osteoarthritis, along with the aforementioned degenerative disc disease and mechanical loading factors endured by the spine, can cause severe lower back pain because of the potential impingement and injury that can happen to the spinal cord in a couple ways such as bulging discs, disc prolapse and protrusion, and finally disc herniation/rupture and extrusion [94].

### 3.3. Bulging Disc

**Figure 11:**
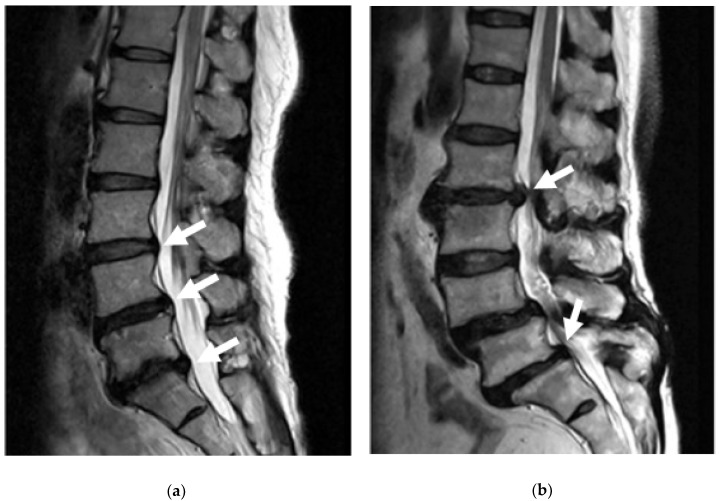
MRI image showing a slight bulge of the annulus into the spinal canal without severe impingement (**a**). MRI image showing a full lumbar disc herniation with substantial spinal stenosis and nerve-root compression (**b**) [97].

Bulging discs are considered the starting stage for problems with impingement to the spine and are generally associated with fatigue failure from mechanical loading and disc degeneration of Grade 0 (negligible degeneration), Grade I, and Grade II [96]. In the early stages of disc degeneration, when the annulus fibrosus starts to dry out and become more fibrous, the amount of mechanical strain it can take decreases. With high compressive loads that are put onto the discs that require the nucleus to push out causing pressure to the annulus, this can cause problems such as small tears part way through the lamellae. When some of these lamellae tear, usually in the posterior section of the disc, the pressure from the nucleus can make the discs bulge outwards due to the lack of support from the annulus, Figure 11 [97]. 

When the disc bulges into the spinal canal, it can put pressure onto the spinal cord and other spinal nerves, one of the most prominent being the sciatic nerve, causing pain and sometimes even numbness [22]. Although the pain from these bulging discs is bearable, if left untreated, they can lead to even more severe problems such as disc herniation.

### 3.4. Disc Herniation (Prolapse/Rupture)

Disc herniation, also referred to as disc prolapse, rupture, and extrusion, occurs in later stages of disc degeneration, Grades III–V, and is brought about by increased mechanical loading and fatigue of the annulus that has typically already started to bulge [96]. As the annulus becomes more and more fibrous with degeneration, there is an increase in tears through the lamellae due to the forces of the nucleus. When the tears penetrate all the way through the annulus, the nucleus starts to push out and leak into the spinal canal, Figure 11 [98]. Unlike bulging discs, because the nucleus actually leaks into the spinal canal, it tends to have much more significant impacts on an individual’s life due to the severe impingement on nerves of the spinal cord, causing pain, numbness, tingling, and weakness [99].

The most common area for disc herniation is in the lumbar spine, particularly in the lower lumbar, with roughly 56% of herniations occurring in the L4/L5 disc and roughly 41% occurring in the L5/S1 disc [99]. Both of these disc herniations can play significant roles in the quality of an individual’s life, since they both are involved with the sciatic nerve. The sciatic nerve, as mentioned in the above anatomy, runs all the way from the lower spine down through the back of the leg. When impinged, this can cause severe problems with motions such as standing up from a seated position, walking, bending over, and twisting of the upper body, and can cause pain, numbness, weakness and general discomfort throughout the entire low extremity. With disc herniation, surgery is very often required to fix it, however with a bulging disc or other lower back pain, some other less invasive procedures exist [100]. 

## 4. Current Treatment Techniques

Depending on the severity of disc degeneration, and whether or not a disc is bulging or herniated, there are multiple treatment options, both invasive (surgical) and noninvasive (nonsurgical). The most common treatments include physical therapy, epidural injections, and medications for noninvasive, and radiofrequency ablation, spinal fusion surgery, synthetic total disc replacements, and annulus fibrosus repair for invasive. Although pain and disability are usually relieved for a period of time, the effectiveness of these treatments are less than ideal, due to certain problems associated with each, further discussed below. Therefore, along with the invasive and noninvasive options, other less-traditional treatments are being researched such as the use of stem cells, growth factors, and gene therapy with the theoretical potential to prevent, slow, or even reverse disc degeneration, as well as tissue engineered scaffolds in order to completely replace degenerated discs [101]. 

### 4.1. Nonsurgical Treatments

#### 4.1.1. Physical Therapy

With disc degeneration, comes lack of support and stability of the spine due to the decreasing biomechanical functions of the intervertebral disc. In order to regain this loss of function, the muscles surrounding the spine and supporting spinal loads must increase in strength and stability, therefore decreasing the need for intervertebral disc support for the spine. A solution to this problem is physical/functional therapy, of which benefits include increased strength, flexibility, and range of motion [102]. Improving motion in a joint is one of the optimal ways to relieve pain. This can be accomplished by stretching and flexibility exercises which improve mobility in the joints and muscles of the spine and extremities. The next is increasing strength with exercises for the trunk muscles, providing greater support for the spinal joints, and arm and leg muscles, reducing the workload required by the spinal joints. Aerobic exercising has also been shown to relieve lower back pain by promoting a healthy body weight and improving overall strength and mobility [102]. Other therapies include deep tissue massaging, posture and movement education for daily life (functional therapy), and special treatments such as ice, electrical stimulation, traction, and ultrasound. Ultrasound treatment, in particular, has been shown to significantly improve lower back pain for individuals suffering from degenerative and even prolapsed discs, although it is only a temporary solution [103]. Physical therapy does not reverse the age-related disc degenerative changes, however, healing should be promoted by stimulating cells, boosting metabolite transport, and preventing adhesions and re-injury, which in turn will relieve pain caused by degenerative disc disease [104]. 

#### 4.1.2. Epidural Steroid Injections

Epidural steroid injections are one of the most common injections for relief of pain, by reducing inflammation caused by degenerative disc disease. The injections consist of cortisone, which has anti-inflammatory properties reducing and further preventing additional inflammation, combined with a local anesthetic, which offers immediate short-term pain relief. Both of these components help to turn off the inflammatory chemicals produced by the body’s immune system that can lead to future flare-ups [105]. It is injected into the epidural space that surrounds the membrane covering the spine and nerve roots. Because it is administered so close to the area of pain, this treatment tends to have better effects and outcomes than that of oral and topical medications, however it can only be performed three times a year due to the negative side effects of the steroids in the body and the effects only last 1–2 months. Also, it does not reverse the changes of degenerative disc disease already caused by aging, with over two-thirds of patients undergoing an additional invasive treatment within two years of the epidural injections [106]. 

#### 4.1.3. Medications

For low to moderate lower back pain caused by degeneration of the discs and spine, oral and topical medications can be prescribed. These medications include over-the-counter acetaminophen (Tylenol) and non-steroidal anti-inflammatory drugs (NSAIDs), anti-depressants, skeletal muscle relaxants, neuropathic agents, opioids (narcotics), and prescription NSAIDs, each having individual and unique benefits depending on the severity and type of pain [107]. 

The acetaminophen and NSAIDs are usually taken for very low, dull chronic pain. Acetaminophen such as Tylenol is used to essentially block the brain’s pain receptors, while NSAIDs such as ibuprofen, naproxen, or aspirin are used to reduce inflammation. The NSAIDs however, need to be taken on a daily basis because they work to build up an anti-inflammatory effect in the immune system [108]. This means that only taking them when pain is present does not work to limit inflammation as well as taking them regularly. Tricyclic anti-depressants are usually given for chronic lower back pain as well. These anti-depressants work similarly to acetaminophen, blocking pain messages on their way to the brain. They also help to increase the body’s production of endorphins, a natural painkiller, and help individuals sleep better, allowing the body to regenerate and recover [107,108]. Skeletal muscle relaxants, such as tizanidine and cyclobenzaprine, are needed for individuals who have acute back pain due to muscle spasms. When their muscles spasm, they put additional stresses onto the discs and spinal nerves causing intense pain through the spine. Neuropathic agents, such as Neurontin and Lyrica, are used when the nerves of the spine are impinged due to a bulging or herniated discs. These medications allow for the specific targeting of nerves to block signals sent to the brain in order to prevent pain. Opioids (narcotics), such as Vicodin and Percocet, are used in extreme cases of spinal pain given their addictive qualities. They work by attaching to receptors in the brain, similar to acetaminophen, however with much higher strength and effect, tending to cause side effects such as slow breathing, general calmness/drowsiness, and an anti-depressant effect. Prescription NSAIDs work exactly the same as over-the-counter NSAIDs, however they tend to work better given their increased strength and potency [107,108]. 

### 4.2. Surgical Treatments

#### 4.2.1. Radiofrequency Ablation

Radiofrequency ablation is a technique that uses heat put through the tip of a needle, either by continuous or pulsed radiofrequency, to denervate an injured disc causing pain to an individual. Nerves of which can be denervated to help with low back pain are the facet nerves, sympathetic nerves, communicating rami, and nerve branches in the disc itself. After anesthesia is administered to the procedure site, a needle or electrode is inserted into the disc or near the small nerve branch, under X-ray, fluoroscopy, computerized axial tomography, or magnetic resonance guidance [109,110]. When in the right position, the tip of the needle or electrode is heated up to the point in which it causes damage or heat lesions to the nerves, destroying them to the point that back pain is relieved. Pain can be relieved usually for 6 to 12 months, and in some cases can last for a few years. It is one of the less invasive operations, and therefore is considered an outpatient surgery, in which the patient is put under local anesthesia and can go home that day without being hospitalized [109,110]. This procedure is usually recommended for patients who have already undergone procedures such as epidural steroid injections, facet joint injections, sympathetic nerve blocks, or other nerve blocks with pain relief lasting shorter than desired. The average cost of this procedure ranges anywhere from $2000 to $5000 based on practitioner, amount of nerves destroyed, and location of spine. If, however degenerative disc disease becomes too severe, this method will not be suitable for long term, and other surgeries or total disc replacements will have to be considered. 

#### 4.2.2. Spinal Fusion Surgery

**Figure 12:**
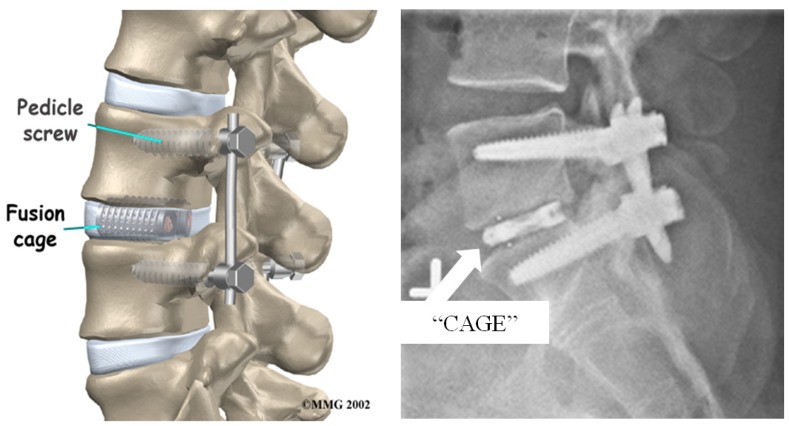
Example image of spinal fusion surgery using titanium cages loaded with hydroxyapatites and pedicle screws and rods to keep stability and anatomic alignment in spinal segment [114].

Spinal fusion surgery has been widely accepted as a useful treatment option for correcting severe disc degeneration disease, however its efficacy and success remain controversial. Multiple approaches for this procedure can be taken such as posterolateral fusion, anterior lumbar interbody fusion, posterior lumbar interbody fusion, and lateral lumbar interbody fusion, each being a minimally invasive technique to lumbar spinal fusion [101]. For this treatment, the damaged disc is completely removed from the spine and replaced with either an osteoconductive-filled titanium cage or a hydroxyapatite bone graft extender that sits in between the two vertebrae [111,112]. Titanium plates are then attached to the vertebrae above and below the titanium cage, using titanium pedicle screws as fasteners, to offer additional support to the spine after surgery, Figure 12. This allows for stability of the spine and correct anatomic alignment of the spinal segments by sharing the loads acting on the spine, until the point in which solid biological fusion occurs into a single bone [113]. This is important because if the adjacent segment motion is altered, it can lead to further degeneration of additional discs and motion segments [101]. Once this occurs, the patient can opt to have the plates and screws removed via another surgery.

Although spinal fusion surgery tends to alleviate discogenic pain associated with degenerative changes, due to eliminating motion between certain vertebrae, some other problems can arise that could potentially be more detrimental in the long run. When two vertebrae are fused together, there becomes no load absorbing center, which severely limits shock absorption and increases loads and stresses on surrounding tissues and discs, as well as limiting mobility [101,113]. This gives way to additional intervertebral disc degeneration in the adjacent levels, which will then potentially need to be fused as well. However, since the lumbar is the main contributor to the mobility of the spine, preserving that mobility is vital to everyday activity. For this reason, most doctors refuse to fuse more than three levels of the spine together so to not hinder the movements of everyday life and cause more problems than leaving the damaged disc in the spine [115]. It is estimated that over 137,000 cervical and 162,000 lumbar spinal fusion surgeries are performed every year in the United States alone, totaling over 325,000 fusions, each costing over $34,000 for the average hospital bill, excluding professional fees and equipment fees [116,117]. In the last few years however, interest in total disc replacement instead of spinal fusion surgery has grown due to their ability to retain motion of the lumbar motion segments [116].

#### 4.2.3. Total Disc Replacement

Total disc replacements (TDR) is a treatment option that consists of the removal of the degenerative native disc and replacing it with a synthetic implant. This option offers the mobility that is required for the lumbar section that spinal fusion surgery does not, however, they are still not as mainstream as fusion surgery [116]. In order for a TDR to be considered effective, the implant must fulfill four main requirements: (1) a solid, nondestructive interface with the adjacent vertebral bodies; (2) provide mobility to mimic the range of motion of the natural disc; (3) resist wear and tear in the body to reduce debris contamination in the body; (4) have the ability to absorb shock and distribute loads evenly and effectively [118]. In all of these requirements, the lumbar spine TDR must perform at a more demanding level than that of the cervical spine due to the extra loads it must bear. Therefore, fabrication of TDRs for the lumbar spine have proven to be much more difficult when compared to those for the cervical spine. Lumbar TDR can be classified according to their configuration, materials, bearing type, and regulatory status, Table 5. The configurations of the TDR devices are designed to maximize the range of motion within the realm of natural disc mobility and permit the most freedom. Each configuration of TDR is dependent upon the type of modules involved in the working disc, therefore current designs are built around a bearing for maximum mobility [118]. The bearing systems used includes one-piece (1P), Metal-on-Metal (MoM), or Metal-on-Polymer (MoP), with MoM and MoP bearings using a ball and socket design to allow for motion in all directions. Only two lumbar disc prostheses have currently been approved for use by the Food and Drug Administration (FDA), the Charite^®^ from DePuy Spine and the Prodisc^®^ L from DePuy Synthes, although many more are becoming prevalent through trial testing such as Maverick^TM^, Kineflex^®^, Freedom^®^, and Mobidisc^®^ [118].

Although there are a lot of different TDR options, each has their disadvantages, with only the two previously mentioned even being FDA approved. Ball and socket bearing systems give way to the possibility of hypermobility within the motion segment, greater amounts of debris from wear, and stress concentration within bearing itself, which causes higher stresses to act on the vertebrae. It has also been shown that these systems show no elastic shock absorption properties, even between MoM and ultra-high molecular weight polyethylene (UHMWPE) cores (MoP) [119]. The one-piece bearing systems were designed to potentially counteract the above flaws by adequately mimicking the natural disc behavior; reducing the number of surfaces on which wear can occur, reducing the hypermobility of the joint, and distributing load and absorbing shock [118,119]. The flaws with the one-piece systems, however, are that the elastomer core used suffers greater chance of material tears either within the material or at the adhesion interface between the different materials. They experience short fatigue life and are still recent designs, needing further evaluation of wear and corrosion resistance [118]. Creep deformations and hysteresis properties of the elastomeric material may be limiting factors as well [119]. Each TDR system experiences failure through two mechanisms of degradation of the implant, wear and corrosion. These degradations are to be expected with articulating bearings in harsh environments, however act more heavily on some materials as opposed to others, Table 6. 

When using MoM devices, the degradation due to wear is minimal when compared to MoP devices and PEEK-on-PEEK devices (PoP), however the toxicity introduced to the body is relatively the same. Although the volume of wear particles might be smaller, the CoCr wear particles are chemically reactive within the body causing corrosion, tribocorrosion, and toxic and biological responses, such as metallosis, biological reactions, osteolysis, and inflammation. When MoP devices wear, the particles produced tend to be fine, needle and fiber-shaped particles which are less chemically reactive than the metal particles although bigger in size. The PoP devices shows properties of resisting expulsion of nucleus particles, and superior fatigue resistance and wear resistance, however severe biological reactions occur causing device rejection and migration of device into surrounding muscle tissue [118]. Each of these systems have their benefits and disadvantages when compared to each other, however when compared to spinal fusion surgery, shows great advantages in the range of mobility. If a disc has undergone some degeneration, but is not yet to the point of spinal fusion or total disc replacement, other actions can be taken such as annulus fibrosus repair.

#### 4.2.4. Repair of Annulus Fibrosus

The annulus fibrosus is involved in almost any pathological condition of the degenerating spine, therefore when its function becomes impaired, plays a fundamental role in two specific clinical situations. It acts as the main source of discogenic low back pain, and as the origin of disc herniation due to its insufficiency caused by degenerative disc disease. As previously discussed, when small fissures occur in the annulus, a repair process takes place in which granulation tissue is formed along with neovascularization and concomitant ingrowth of nerve fibers. This causes chronic discogenic pain throughout the disc due to the pressure being sustained by the nerves. Annulus fibrosus repair is the procedure to fix those tears before the disc herniates, and is usually performed in relatively young patients with very minor degenerative changes. Efficient annulus repair could significantly limit the need for future surgeries in certain cases in which there is potential of disc herniation, however no herniation has currently occurred. When the ruptures are treated, the focus is on improving cell-biomaterial interaction, using an initial implant to provide immediate closure of the tear and maintain mechanical properties of the disc, while the cellular component starts the regenerative process within the disc. This process, however, is not complete or satisfactory when it comes to being a permanent solution, but instead is a preventive measure for disc prolapse [120]. The most straight-forward solution is suturing the annulus tear shut, helping give the disc a stronger tendency to heal itself. However, its sole purpose is the containment of the nucleus pulposus and does not compensate for the loss of the annulus nor reverse the biomechanical changes [121]. One way to adjust for the lack of compensation could be the addition of growth factors in order to enhance the regenerative process of the annulus tissue [122]. Vadala et al. studied the potential of Transforming Growth Factor-β (TGF-β) loaded microfibrous poly(L-lactide) scaffold in vitro. The biological evaluation of the scaffolds was performed using bovine annulus fibrosus cells that were cultured on the scaffold for up to three weeks [122]. These electrospun scaffolds allowed for the closure of the defect site while releasing the TGF-β, inducing an anabolic stimulus on the annulus cells, mimicking the ECM environment of the tissue [122]. The scaffolds, together with the TGF-β release, promoted rapid cell growth compared to the control, resulting in the deposition of significantly greater amounts of GAGs and total collagen within the annulus tissue, as well as a higher neo-ECM thickness [122]. Another method studied by Cruz et al., focuses on the repair of annulus fibrosus defects through a cell-seeded adhesive biomaterial, further detailed in Section 5 [123]. 

Annulus fibrosus repair gives great advantages to those who have yet to have a full disc herniation, as well as those only experiencing minimal degeneration, giving them the opportunity to forgo the potential chance for surgery in the future. It should be noted however, that this treatment option is not a cure for degenerative disc disease, but a preventative measure taken to increase the longevity of the native disc, potentially permanently, depending on an individual’s particular life style.

## 5. Tissue Engineering and Regeneration Strategies

With all of these options facing difficult challenges, tissue engineering and regenerative strategies stand out as potential solutions. These include some form of gene therapy, regeneration strategies via delivery of bioactive molecules, e.g., growth factors, or a material scaffold with or without cells. Gene therapy and regeneration with growth factors, cells, or enzymes, such as ADAMTS5, have been researched in rats for early stage trials, showing greater GAGs and total collagen deposition for the TGF-β1 treated animals, and successful suppression of the degradation of the nucleus pulposus for ADAMTS5 treated ones [122,124]. Along with these, a similar approach was studied by Guterl et al. to target caspase 3 in rabbit models, in order to disrupt the execution of apoptosis [125]. A direct injection of Alexa Fluor 555-caspase 3 small interfering RNA (siRNA) into the rabbit intervertebral disc was used to determine the effect on suppression of degenerative changes within the disc. Compared to the caspase 3 siRNA control, the Alexa Fluor 555-caspase 3 siRNA resulted in a significant decrease in serum-starved apoptotic cells, as well as a significant suppression of the degenerative changes to the disc [125]. 

In more current regeneration efforts, FDA-cleared Phase III adult stem cells were used in a test study to treat chronic lower back pain associated with degenerative disc disease. The use of mesenchymal precursor cells directly injected into the lumbar disc will hopefully show some ability to regenerate lost tissue of the disc [126]. Another system studied by Alini et al. uses implanted intervertebral disc cells in a scaffold of collagen and hyaluronan, or entrapped into a chitosan gel, with either fetal calf serum (FCS) or growth factors (TGF-β1, bFGF, and IGF-1) to modulate ECM synthesis [124]. The FCS and TGF-β1 were able to induce proteoglycan synthesis, while the presence of bFGF and IGF-1 reduced proteoglycan synthesis. However, the IGF-1 was shown to stimulate cell division by the greatest extent [124]. By day 20 of the culture, in FCS and the varying growth factors, not only did the matrices contain aggrecan, but also other small leucine-rich repeat proteoglycan found in the normal disc and both collagen type I and II [124]. Although all proteoglycans found in a normal disc were synthesized, the construct was not able to retain the majority of its proteoglycans, resulting in the inability to withstand the compressive loads normally subjected to an intervertebral disc [124]. Another future direction is to look more into the regeneration and repair of the annulus tissues as opposed to the nucleus tissues. Efforts for novel therapies have mainly been directed towards nucleus tissue regeneration and replacement, however a main challenge is the development of strategies and techniques that deal with the degenerated annulus, preferably in a combined approach with the nucleus [121]. A recent study performed by Cruz et al. shows the possibility to help repair damaged annulus fibrosus tissue through a cell-seeded adhesive biomaterial [123]. Multiple genipin-crosslinked fibrin adhesive cell carriers were developed with varying genipin to fibrin ratios, to determine the optimal composition for mimicking natural annulus fibrosus tissue. Among the adhesive cell carrier, were encapsulated bovine annulus fibrosus cells to show the feasibility of cell delivery to the injured tissue [123]. The cell-seeded adhesive demonstrated shear and compressive properties matching those of the annulus fibrosus tissue, while significantly improving failure strength in situ. As well, the adhesive showed increased cell viability and GAGs production [123]. These efforts could propel the future of annulus repair, offering successful preventative methods, as opposed to perpetuating the need for herniation surgeries and issues associated with them.

In the last few years, tissue engineered scaffolds for total intervertebral disc replacement have risen to the forefront of current biomaterial literature and research in order to address the challenges mentioned above [123,127,128,129,130,131,132,133,134,135,136,137,138,139,140,141,142,143,144]. Scaffolds have been fabricated using both natural and synthetic materials, as well as most containing embedded cells for natural tissue growth and integration [127]. Two recent studies by Iu et al. and Yang et al. focus on total intervertebral disc replacement using a hierarchically organized annulus fibrosus and a hydrogel-like nucleus pulposus, however differ with respect to materials, procedures, and objectives [129,130]. Iu et al. focuses on an in vitro generated intervertebral disc with the ability of tissue integration between the fabricated annulus and nucleus to better mimic the natural disc [129]. The annulus fibrosus was created using six lamellae comprised of aligned nanofibrous polycarbonate urethane scaffolds cultured with annulus fibrosus cells in a Dulbecco’s modified Eagle’s medium (DMEM) with 20% fetal bovine serum (FBS) for three weeks to produce an integrated type I collagen-rich ECM [129]. This surrounded the nucleus pulposus tissue comprised of a type II collagen- and aggrecan-rich ECM hydrogel which was cultured with nucleus pulposus cells in DMEM with 20% FBS solution for four weeks [129]. Both tissues were then combined and co-cultured to create the full intervertebral disc model with integration between the tissues [129]. It was shown that this system successfully integrated the annulus fibrosus lamellae not only to the nucleus tissue, but also to each other, allowing for interlamellar connectivity. When biologically and mechanically tested both in vitro and in vivo, the tissue engineered intervertebral disc showed no inflammatory reaction and was able to stand up to the interlamellar and annulus-nucleus interface shear forces experienced by the disc in the spine [129]. The in vitro studies demonstrated the possibility to create an intervertebral disc with mechanically stable tissue integration that was able to grow similar ECMs as the natural tissue. The in vivo studies demonstrated the ability of the engineered nucleus pulposus to form tissue in vivo, as well as test the disc’s ability to develop intradiscal swelling pressure under load [129]. However, this experiment was evaluated using a bovine caudal spine rather than a human spine, resulting in a smaller intervertebral disc model, and would therefore need to pursue further research to evaluate the scalability and suitability of the system for human biological disc replacement [129]. Yang et al., on the other hand, focused on creating a total intervertebral disc replacement that can integrate natural tissue in vivo, and demonstrates excellent hydrophilicity and functional performance [130]. The hydrophilicity property of this scaffold is highly important for not only the swelling properties of the disc for mechanical stability, but also diffusion of nutrients through the disc for cell viability. The annulus fibrosus mimicry was fabricated using electrospun polycaprolactone/poly(d,l-lactide-*co*-glycolide)/collagen type I nanofibers to create a hierarchically organized, concentric ring-aligned structure, and the nucleus pulposus mimicry was fabricated using an alginate hydrogel [130]. Both components were cultured for 3 days in a DMEM/F12, 10% FBS, and 1% penicillin-streptomycin with a seeded cell density of 2500 cells/cm, and tested for biocompatibility and mechanical integrity before being implanted into rat caudal spine models [130]. In vivo, the replacement discs demonstrated excellent hydrophilicity, mimicking the highly hydrated native tissue, as well as shape maintenance, integration with surrounding natural tissue, acceptable mechanical support, and flexibility [130]. This study shows the potential of scaffold materials as intervertebral disc tissue engineering and regeneration platforms in vivo. However, like the previous study, the fabricated disc was smaller than that of a human, since it was synthesized for a rat model, resulting in the need for much lower mechanical properties for the materials. Therefore, further research would be needed to scale this approach to human trials [130].

Bhunia et al. studied a method for correcting degenerative disc disease that lies between annulus fibrosus repair and total intervertebral disc replacement [131]. Bhunia et al. focused on the recapitulation of form and function of the intervertebral disc through a silk protein-based multilayered, disc-like angle-ply annulus fibrosus scaffold comprising of multiple concentric lamellae. The scaffold was fabricated to resemble the hierarchical structure of the natural tissue, which was verified through electron microscopy [131]. These “biodiscs” demonstrated mechanical properties similar to those of the native tissue, as well as support of human mesenchymal stem cell proliferation and differentiation, and deposition of a sufficient amount of ECM after 14 days of culture. A section of the biodisc was implanted subcutaneously in a mice model and retrieved after one and four weeks of implantation, showing negligible immune response [131]. However, the proposed system lacked the ability to replace the entirety of the intervertebral disc, leading to the need for further research with the addition of an implantable nucleus. A recent study by Ghorbani et al. shows a promising method for nucleus pulposus replacement utilizing an injectable hydrogel [132]. The hydrogel was comprised of chitosan-β-glycerophosphate-hyaluronic acid, chondroitin-6-sulfate, type II collagen, gelatin, and fibroin silk, in order to replicate the complexity of the natural nucleus pulposus ECM. The synthesized nucleus demonstrated ideal hydrophilicity, stability, and strength when subjected to loads, with the storage modulus remaining nearly constant over a wide range of strain. In vitro tests were conducted using MTT and trypan blue to quantify and qualify cell growth and cytotoxicity, revealing the hydrogel to be cytocompatible with good cell attachment and growth [132]. Like the study performed by Bhunia et al., the solution only focuses on nucleus replacement/regeneration, therefore further research is needed with the combination of a tissue engineered annulus fibrosus.

As pointed out earlier, many of these studies use relatively weak electrospun scaffolds or combinations thereof with hydrogels that lead to mechanical properties that are on the range of rat native IVD but that are far from recapitulating those of human IVD. Recent studies have focused their attention on the development of sophisticated scaffolds and materials that can better mimic the outstanding mechanical properties of human IVD (Table 7). Novel materials and composites on the form of hydrogels have been investigated for the replacement of the nucleus pulposus. These include interpenetrating networks based on dextran, gelatin and poly (ethylene glycol) [133]; cross-linked collagen-II, aggrecan and hyaluronan [134]; and silk-fibrin and hyaluronic acid composite hydrogels [135], among others. For the annulus fibrosus scaffolds on the shape of fibrous matts or polymer films are generally preferred, mimicking the structure of the native tissue. To this end, novel materials have also been investigated such as nanocellulose reinforced gellan-gum hydrogels [136]; electrospun aligned polyurethane scaffolds or poly(trimethylene carbonate) structures prepared by lithography and covered with a polyester urethane membrane [137], among others [138]. However, these studies report on the fabrication of individual tissues rather than the recapitulation of the whole organ, what makes difficult the extrapolation of these results to a more complete and practicable approach.

Hu et al. recently reported on the fabrication of 3D printed scaffolds based on the combination of poly (lactic acid) (PLA) and gellan gum-poly (ethylene glycol) diacrylate (GG-PEGDA) double network hydrogel [139]. This combination allowed fine tuning of the mechanical properties of the overall organ by changing the infill patterns and the density of the PLA framework. Initial studies with in-situ bioprinted human mesenchymal stem cells (hMSCs) show good cell viability and spreading within the constructs. Although this first study shows an interesting 3D printing approach, the final scaffold represents a rather homogeneous construct and not the clearly compartmented native organ. Yang et al. overcame this issue by designing and fabricating a triphasic scaffolds that aimed at recapitulating the three main structures of native IVD, the nucleus pulposus and the inner and outer rings of the annulus fibrosus, while targeting the mechanical properties of human IVD [140]. The authors used a chitosan hydrogel to mimic the inner nucleus pulposus that was then surrounded by a poly(butylene succinate-co-terephthalate) (PBST) fiber film and a poly(ether ether ketone) (PEEK) ring to mimic the inner and outer annulus fibrosus, respectively. This multi-layered structure was seeded with porcine IVD cells and used on an in vivo porcine spine model. After 4 and 8 weeks of implantation the cell-scaffold construct retained its original height and showed a histological gross appearance that resembled that of the native tissue. Moreover, the compressive Young’s modulus of the construct was 58.4 ± 12.9 MPa, similar to that measured for the native tissue (71.5 ± 18.2 MPa) [140]. Under a similar concept, Choy et al. studied the potential of biphasic scaffolds for full IVD tissue engineering. They developed a collagen and GAG hydrogel core that was encapsulated on a multiple lamella of photochemically cross-linked collagen membranes, mimicking the nucleus pulposus and the annulus fibrosus, respectively [141]. These constructs were capable of recovering up to 87% their original size after compression and showed a dynamic mechanical stiffness similar of that of the native rabbit IVD. Although this studied showed great promise in terms of mechanical properties and shape recovery of the constructs, a detailed biological study is still missing [141].

Other studies, such as those by Hudson et al., have focused more on the adaptation of tissue engineered intervertebral discs when exposed to certain environments and conditions [142,143]. In both studies, the intervertebral disc was fabricated by floating an injection molded alginate hydrogel nucleus pulposus in a collagen type I annulus fibrosus that contracted around the nucleus given ample time. This study showed that hypoxic expansion of human mesenchymal stem cells enhances the maturation of the tissue engineered disc, as opposed to normoxic environments [142]. Hypoxic conditions, which correlated to 1 to 5% oxygen content, resulted in an increase in ECM production, as well as driving chondrogenesis of the embedded stem cells, when compared to normoxic conditions (21% oxygen). Also, the hypoxic discs were stiffened up to 141%, and showed an increase in GAGs and collagen content within the nucleus, compared to normoxic [142]. The results obtained in this study show the benefit of hypoxic maturation of stem cells within the tissue engineered disc before implantation, however, to fully grasp the effectiveness of this scaffold, in vivo tests will need to be performed. Another study focused on the potential of dynamic unconfined compressive loading on the tissue regeneration/deposition rate [143]. Each tissue engineered disc was subjected to mechanical stimulation from a strain amplitude range of 1–10% for two weeks with a cycle of one hour on, one hour off, one hour on. The discs were then evaluated for biochemical and mechanical properties, which showed an increase in GAGs and hydroxyproline content, and equilibrium and instantaneous modulus for both the nucleus and annulus [143]. These results suggest that dynamic loading increases the functionality of the tissue engineered disc, with each section experiencing region dependent responses, which could be used to expedite maturation for implantation. Although promising, further research would need to be performed in vivo as well as on a larger scale models, bearing a closer resemblance to the natural intervertebral disc [143].

Altogether, these studies show the need of further development and study of materials and scaffolds fabrication techniques for the regeneration of full IVD. The outstanding mechanical properties and complexity of the multi-phasic structure of the native organ will require the development of also complex systems that can recapitulate these features.

## 6. Conclusions

Although great strides have been made in the field of degenerative disc disease, there is still a lot more progress to be made, given the challenges faced with every treatment option currently available. In early stages of degenerative disc disease, noninvasive treatments or treatments such as radiofrequency ablation and annulus fibrosus repair can be of great help, however they only mitigate the symptoms instead of the actual cause. Noninvasive treatments face the challenges of only dealing with some of the symptoms of the pain rather than dealing with the actual degeneration of the discs, therefore allowing the discs to continue to degrade to the future point of needing invasive treatments. Radiofrequency, although good for reducing pain, has the challenge of only lasting short term, a few months to a year in most cases. Also, it is an expensive procedure that has to be repeated every six months [109]. Annulus repair seems to be a better option for young adults with degeneration to the point just before herniation to significantly reduce the need for future surgery, but faces challenges of, again, only fixing the symptoms of the main problem as well as not being to mend any biological changes/losses within the annulus [120]. When disc degeneration gets even worse, greater procedures need to take place, such as spinal fusion surgery and TDR. Spinal fusion surgery is, as of today, the most common life-long solution to severe disc degeneration, however it is struck with multiple challenges such as significantly limiting mobility and adding additional stresses to the adjacent motion segments potentially causing greater degeneration in other intervertebral discs [113]. TDR has been shown to help retain the mobility that spinal fusion cannot, but can sometimes lead to hypermobility of the joint, can wear and corrode causing a biological reaction in the body, and more often than not, does not distribute load nor absorb shock, but rather transfers it directly into the adjacent vertebrae [118]. These challenges have led to vast research in the field of tissue engineering for disc degeneration. Even though scaffolds for disc regeneration are taking strides in the right direction, many still remain in a premature state. Each have their own benefits, but also complications, including scalability, tunability, tissue integration, or optimal mechanical properties. Therefore, the gap between the translation of this research to the clinic still remains fairly large with many hurdles to overcome, leading to the need for future research [127,144]. With degenerative disc disease posing such a large problem for individuals and society, and no current ideal treatment options that come without complications, there is a welcoming for future research in the field of tissue engineered biomaterials for the solution of total intervertebral disc replacement [128,129,130,131,132,133,134,135,136,137,138,139,140,141,142,143,144].

## Figures and Tables

**Table 1:** Average dimensions of the intervertebral discs in the cervical, thoracic, and lumbar spine [29,30,31,32,33].

**Cervical IVD Dimensions**	**C2/C3**	**C3/C4**		**C4/C5**		**C5/C6**		**C6/C7**		**C7/T1**
**Area (mm^2^)**	190 ± 10	280 ± 40		240 ± 20		300 ± 30		460 ± 5		440 ± 5
**Thickness (mm)**	3.51 ± 0.71	3.74 ± 0.36		4.07 ± 0.36		4.45 ± 0.21		4.11 ± 0.28		4.50 ± 0.53
**Thoracic IVD Dimensions**	**T1/T2**	**T2/T3**		**T3/T4**		**T4/T5**		**T5/T6**		**T6/T7**
**Area (mm^2^)**	510 ± 50	490 ± 5		485 ± 5		450 ± 40		605 ± 20		750 ± 10
**Thickness (mm)**	4.40 ± 0.65	3.50 ± 0.69		3.30 ± 0.50		3.20 ± 0.47		3.50 ± 0.47		4.10 ± 0.47
**Thoracic IVD Dimensions**	**T7/T8**	**T8/T9**		**T9/T10**		**T10/T11**		**T11/T12**		**T12/L1**
**Area (mm^2^)**	710 ± 30	900 ± 10		840 ± 30		1080 ± 20		1170 ± 30		1190 ± 40
**Thickness (mm)**	3.90 ± 0.72	5.30 ± 0.80		4.80 ± 1.07		6.50 ± 0.97		5.40 ± 0.95		6.8 ± 0.21
**Lumbar IVD Dimensions**	**L1/L2**		**L2/L3**		**L3/L4**		**L4/L5**		**L5/S1**	
**Area (mm^2^)**	1400 ± 20		1640 ± 50		1690 ± 40		1660 ± 30		1680 ± 30	
**Thickness (mm)**	7.65 ± 0.57		8.90 ± 0.25		9.25 ± 0.29		9.90 ± 0.49		9.35 ± 1.06	

(From References [29,30,31,32,33]). Cervical disc thicknesses were taken from 19 Chinese cadaveric humans of no specified age or gender. Standard deviations were estimated from graphical error bars. Thoracic disc thicknesses were taken from 15 healthy female and male cadaveric humans with average ages of 58.67 ± 10.74 years and 56.20 ± 11.65 years, respectively. Lumbar disc thicknesses were taken from 607 female and 633 male human spines with age ranges from 20–92 years and 20–87 years, respectively. Standard deviations were estimated from graphical error bars. All of the cross-sectional areas were taken from 4 full human cadaver spines of the following demographics: Male of 73 years, female of 86 years, female of 85 years, and female of 80 years.

**Table 2:** Types of collagen found in lamellae of the annulus fibrosus [42,43,44].

Collagen Type	Structure	Genes	Alpha Chains	% Collagen Distribution
**Collagen I**	Large diameter, 67-nm banded fibrils	COL1A1 COL1A2	α1(I) α2(I)	Increases from 0→100 from inner to outer regions
**Collagen II**	67-nm banded fibrils	COL2A1	α1(II)	Decreases from 100→0 from inner to outer regions

**Table 3:** Mechanical properties of the annulus fibrosus and nucleus pulposus [50,52,53,54,55].

**Tensile Properties of the Annulus Fibrosus**								
**Sample**	**Sample Specification**	**Ultimate Stress, MPa**		**Elastic Modulus, MPa**	**Yield Strain, %**		**Ultimate Strain, %**	**Stiffness, N/m**
**Bulk Annulus**	**Outer, A**	3.9 ± 1.8		16.4 ± 7.0	20–30 *		65 ± 16	5.7 ± 3.4
	**Outer, P**	8.6 ± 4.3		61.8 ± 23.2	20–30 *		34 ± 11	5.7 ± 3.4
	**Inner**	0.9		--	20–30 *		33	1.2 ± 1.1
**Single Lamella**	**Parallel**	--		80–120	--		--	--
	**Perpendicular**	--		0.22	--		--	--
**Compressive Properties of the Annulus Fibrosus**								
**Section**	**Swell Pressure, (P_sw_), MPa**		**Modulus, (H_A_), MPa**			**Permeability, (k), (×10^−15^ m^4^/N-s)**		
**Anterior**	0.11 ± 0.05		0.36 ± 0.15			0.26 ± 0.12		
**Posterior**	0.14 ± 0.06		0.40 ± 0.18			0.23 ± 0.09		
**Outer**	0.11 ± 0.07		0.44 ± 0.21			0.25 ± 0.11		
**Middle**	0.14 ± 0.04		0.42 ± 0.10			0.22 ± 0.06		
**Inner**	0.12 ± 0.04		0.27 ± 0.11			0.27 ± 0.13		
**Compressive Properties of the Nucleus Pulposus**								
**Sample**	**Swell Pressure, (P_sw_), MPa**		**Modulus, (H_A_), MPa**			**Permeability, (k), (×10^−16^ m^4^/N-s)**		
**Nucleus Pulposus**	0.138		1.0			9.0		

A/P, anterior/posterior section of the annulus. Parallel/Perpendicular, alignment of testing in relation to the fiber orientation. * Only one value was ascertained for entirety of the annulus fibrosus. Tensile properties for the bulk annulus fibrosus were taken from 7 cadaveric human lumbar spines. Tensile properties for the single lamella were taken from 8 male and 3 female cadaveric human lumbar spines with an average age of 57.9 ± 15.4 years. The spines were harvested within 24 h of death. Compressive properties of the annulus fibrosus were taken from cadaveric humans of no specified age or gender. Compressive properties of the nucleus pulposus were taken from 10 IRB-approved cadaveric human lumbar spines with ages between 19–80 years (average of 57.5 years) and of no specified gender.

**Table 4:** Distinction between different grades of disc degeneration based on magnetic resonance imaging (MRI) scans [87].

Grade	Structure	Distinction of Nucleus and Annulus	Signal Intensity	Height of Intervertebral Disc
I	Homogenous, bright white	Clear	Hyperintense, isointense to cerebrospinal fluid	Normal
II	Inhomogeneous with or without horizontal bands	Clear	Hyperintense, isointense to cerebrospinal fluid	Normal
III	Inhomogeneous, gray	Unclear	Intermediate	Normal to slightly decreased
IV	Inhomogeneous, gray to black	Lost	Intermediate to hypointense	Normal to moderately decreased
V	Inhomogeneous, black	Lost	Hypointense	Collapsed disc space

**Table 5:** Summary of current total disc replacement (TDR) classification, materials, bearing type, and regulatory status [118].

Device	Classification	Biomaterials	Bearing Design	Examples of Manufacturer
CHARITE	MoP	CoCr-UHMWPE	Mobile	DePuy Spine
Prodisc-L	MoP	CoCr-UHMWPE	Fixed	DePuy Synthes
Activ-L	MoP	CoCr-UHMWPE	Mobile	Aesculap
Mobidisc	MoP	CoCr-UHMWPE	Mobile	LDR Medical
Baguera	MoP	DLC coated Ti-UHMWPE	Fixed	Spineart
NuBlac	PoP	PEEK-PEEK	Fixed	Pioneer
Maverick	MoM	CoCr-CoCr	Fixed	Medtronic
Kineflex	MoM	CoCr-CoCr	Mobile	SpinalMotion
Flexicore	MoM	CoCr-CoCr	Constrained	Stryker
XL-TDR	MoM	CoCr-CoCr	Fixed	NuVasive
CAdisc-L	1P	PU-PC graduated modulus	1P	Rainier Technology
Freedom	1P	Ti plates; silicone PU-PC core	1P	Axiomed
eDisc	1P	Ti plates; elastomer core	1P	Theken
Physio-L	1P	Ti plates; elastomer core	1P	NexGen Spine
M6-L	1P	Ti plates; PU-PC core with UHMWPE fiber encapsulation	1P	Spinal Kinetics
LP-ESP (elastic spine pad)	1P	Ti endplates; PU-PC coated silicone gel with microvoids	1P	FH Orthopedics

CoCr—Cobalt-chromium alloy. UHMWPE—Ultra-high molecular weight polyethylene. DLC—Diamond-like carbon. Ti—Titanium. PEEK—Polyether ether ketone. PU-PC—Polyurethane-polycarbonate elastomer.

**Table 6:** Common problems of different implant materials and their effects leading to failure [118].

Bearing Type	Material	Problems	Effects
Ball and Socket	CoCr	Reactive wear ions and fibrous particles	Metal sensitivity reactions, Inflammation, Osteolysis
		Metallosis	--
		No shock absorption	Compressive stresses on vertebral bodies
	UHMWPE	Large wear volume and wear debris	Bone resorption, Osteolysis
		Plastic deformation	--
		Increased range of motion (hypermobility)	Facet and ligament loading
		No shock absorption	Compressive stresses on vertebral bodies
	PEEK	Prosthesis migration	Biomechanical incompatibility, Stress on remaining annulus, Total rejection of device
		Endplate reaction	Severe biological rejection
1P	PUPC	More studies necessary	

Note: The effects stated are correlated to the problems directly next to it.

**Table 7:** Materials, scaffold architecture, mechanical properties, and cell types used in tissue engineering approaches for IVD.

Targeted Tissue	Material	Structure	Mechanical Properties	Cells	Comments	Reference
Total IVD	AF: Poly caprolactone urethane NP: Collagen II and aggrecan	AF: Nanofibrous, aligned NP: Hydrogel	Compressive modulus of 17.2 ± 7.5 kPa	AF and NP cells	Integration between the two compartments. Tested in vitro and in vivo in a bovine model	[129]
Total IVD	AF: Polycaprolactone/poly(d,l-lactide-*co*-glycolide)/collagen type I NP: Alginate	AF: Electrospun nanofibers to create a concentric ring-structure NP: Hydrogel	Tensile Young’s modulus of 380 MPa	Rat AF and NP cells	Integration with host tissue and between compartment in in vivo rat caudal spine model	[130]
AF	Silk	Concentric layers of lamella sheets on an angle-ply construct	499.18 ± 86.45 kPa	Porcine AF cells and human MSCs	Subcutaneous implantation in rat showed negligible immune response	[131]
NP	chitosan-β-glycerophosphate-hyaluronic acid, chondroitin-6-sulfate, type II collagen, gelatin, and fibroin silk	Hydrogel	≈50 Pa	Rabbit NP cells	Preliminary study with in vitro cell compatibility assays	[132]
IVD	PLA and GG_PEGDA	3D printed	Compressive Young’s modulus of ≈400 MPa	hMSCs	Preliminary study on cell viability	[133]
IVD	NP: Chitosan; inner AF: PBST and outer AF: PEEK	NP: hydrogel and AF fiber film and ring	Compressive Young’s modulus of 58.4 ± 12.9 MPa	Porcine IVD cells	In vivo implantation on a porcine spine model	[134]
NP	Dextran, gelatin and poly (ethylene glycol);	Hydrogel	Compressive Young’s modulus of 15.86 ± 1.7 kPa	Porcine NP cells	In vivo subcutaneous implantation in Lewis rats	[135]
NP	Cross-linked collagen-II, aggrecan and hyaluronan	Hydrogel	Storage modulus of ≈1.25 kPa	Bovine NP cells	7 days in vitro cell culture studies	[136]
NP	Silk-fibrin and hyaluronic acid composite hydrogels	Hydrogel	Compressive modulus of ≈5–7 kPa	Human primary chondrocytes	Full in vitro study with up to 4 weeks cell culture	[137]
AF	Nanocellulose reinforced gellan-gum hydrogels	Hydrogel	Compressive modulus of ≈45–55 kPa	Bovine AF cells	Preliminary in vitro studies	[138]
AF	Electrospun aligned polyurethane scaffolds	Fibrous scaffold	N/A	Rabbit AF derived progenitor cells	7 days in vitro cultures	[139]
AF	poly(trimethylene carbonate) and polyester urethane	Fibrous scaffold	Yield strength of 4.9 ± 1.4 MPa	Human MSCs	In vitro bovine caudal spine organ culture model with or without dynamic load.	[140]

NP: nucleus pulposus and AF: annulus fibrosus.

## References

[1] Britannica T.E.o.E. (2014). Vertebral Column. Encyclopaedia Britannica.

[2] Kibler W.B., Press J., Sciascia A. (2006). The role of core stability in athletic function. Sports Med..

[3] Agur A.M.R., Dalley A.F. (2009). Grant’s Atlas of Anatomy.

[4] Vertebral Column. https://www.kenhub.com/en/start/c/vertebral-column.

[5] Bogduk N., Mercer S. (2000). Biomechanics of the cervical spine. I: Normal kinematics. Clin. Biomech..

[6] Swartz E.E., Floyd R.T., Cendoma M. (2005). Cervical spine functional anatomy and the biomechanics of injury due to compressive loading. J. Athl. Train..

[7] Panjabi M.M., Crisco J.J., Vasavada A., Oda T., Cholewicki J., Nibu K., Shin E. (2001). Mechanical properties of the human cervical spine as shown by three-dimensional load-displacement curves. Spine.

[8] Caridi J.M., Pumberger M., Hughes A.P. (2011). Cervical radiculopathy: A review. HSS J..

[9] Yeung J.T., Johnson J.I., Karim A.S. (2012). Cervical disc herniation presenting with neck pain and contralateral symptoms: A case report. J. Med. Case Rep..

[10] Edmondston S.J., Singer K.P. (1997). Thoracic spine: Anatomical and biomechanical considerations for manual therapy. Man. Ther..

[11] Son E.S., Lee S.H., Park S.Y., Kim K.T., Kang C.H., Cho S.W. (2012). Surgical treatment of t1-2 disc herniation with t1 radiculopathy: A case report with review of the literature. Asian Spine J..

[12] Goh S., Tan C., Price R.I., Edmondston S.J., Song S., Davis S., Singer K.P. (2000). Influence of age and gender on thoracic vertebral body shape and disc degeneration: An MR investigation of 169 cases. J. Anat..

[13] Cervero F., Tattersall J.E. (1986). Somatic and visceral sensory integration in the thoracic spinal cord. Prog. Brain Res..

[14] Boszczyk B.M., Boszczyk A.A., Putz R. (2001). Comparative and functional anatomy of the mammalian lumbar spine. Anat. Rec..

[15] Troup J.D., Hood C.A., Chapman A.E. (1968). Measurements of the sagittal mobility of the lumbar spine and hips. Ann. Phys. Med..

[16] Haughton V.M., Rogers B., Meyerand M.E., Resnick D.K. (2002). Measuring the axial rotation of lumbar vertebrae in vivo with MR imaging. Am. J. Neuroradiol..

[17] Granhed H., Jonson R., Hansson T. (1987). The loads on the lumbar spine during extreme weight lifting. Spine.

[18] Tan S.H., Teo E.C., Chua H.C. (2004). Quantitative three-dimensional anatomy of cervical, thoracic and lumbar vertebrae of Chinese Singaporeans. Eur. Spine J..

[19] Crawford R.P., Cann C.E., Keaveny T.M. (2003). Finite element models predict in vitro vertebral body compressive strength better than quantitative computed tomography. Bone.

[20] Shah J.S., Hampson W.G., Jayson M.I. (1978). The distribution of surface strain in the cadaveric lumbar spine. J. Bone Jt. Surg..

[21] Bogduk N. (1983). The innervation of the lumbar spine. Spine.

[22] Luoma K., Riihimaki H., Luukkonen R., Raininko R., Viikari-Juntura E., Lamminen A. (2000). Low back pain in relation to lumbar disc degeneration. Spine.

[23] Nygaard O.P., Mellgren S.I. (1998). The function of sensory nerve fibers in lumbar radiculopathy—Use of quantitative sensory testing in the exploration of different populations of nerve fibers and dermatomes. Spine.

[24] Takahashi I., Kikuchi S., Sato K., Sato N. (2006). Mechanical load of the lumbar spine during forward bending motion of the trunk—A biomechanical study. Spine.

[25] Bogduk N. (2012). Clinical and Radiological Anatomy of the Lumbar Spine.

[26] Koes B.W., van Tulder M.W., Peul W.C. (2007). Diagnosis and treatment of sciatica. BMJ.

[27] Lirette L.S., Chaiban G., Tolba R., Eissa H. (2014). Coccydynia: An overview of the anatomy, etiology, and treatment of coccyx pain. Ochsner J..

[28] Humzah M.D., Soames R.W. (1988). Human intervertebral disc: Structure and function. Anat. Rec..

[29] Pooni J.S., Hukins D.W., Harris P.F., Hilton R.C., Davies K.E. (1986). Comparison of the structure of human intervertebral discs in the cervical, thoracic and lumbar regions of the spine. Surg. Radiol. Anat..

[30] Mahendra K.A., Rajani J.A., Shailendra J.S., Narsinh H.G. (2015). Morphometric study of the cervical intervertebral disc. Int. J. Anat. Phys. Biochem..

[31] Kunkel M.E., Herkommer A., Reinehr M., Bockers T.M., Wilke H.J. (2011). Morphometric analysis of the relationships between intervertebral disc and vertebral body heights: An anatomical and radiographic study of the human thoracic spine. J. Anat..

[32] Shao Z., Rompe G., Schiltenwolf M. (2002). Radiographic changes in the lumbar intervertebral discs and lumbar vertebrae with age. Spine.

[33] Twomey L., Taylor J. (1985). Age changes in lumbar intervertebral discs. Acta Orthop. Scand..

[34] Davis H. (1994). Increasing Rates of Cervical and Lumbar Spine Surgery in the United-States, 1979–1990. Spine.

[35] Williams M.P., Cherryman G.R., Husband J.E. (1989). Significance of thoracic disc herniation demonstrated by MR imaging. J. Comput. Assist. Tomogr..

[36] Adams M.A., Roughley P.J. (2006). What is intervertebral disc degeneration, and what causes it?. Spine.

[37] Raj P.P. (2008). Intervertebral disc: Anatomy-physiology-pathophysiology-treatment. Pain Pract..

[38] Adams M.A., Derby B., Akhtar R. (2015). Intervertebral Disc Tissues. Mechanical Properties of Aging Soft Tissues.

[39] Galante J.O. (1967). Tensile properties of the human lumbar annulus fibrosus. Acta Orthop. Scand..

[40] Guerin H.L., Elliott D.M. (2007). Quantifying the contributions of structure to annulus fibrosus mechanical function using a nonlinear, anisotropic, hyperelastic model. J. Orthop. Res..

[41] Smith L.J., Fazzalari N.L. (2009). The elastic fibre network of the human lumbar anulus fibrosus: Architecture, mechanical function and potential role in the progression of intervertebral disc degeneration. Eur. Spine J..

[42] Ricard-Blum S. (2011). The Collagen Family. CSH Perspect. Biol..

[43] Eyre D.R., Muir H. (1976). Types I and II collagens in intervertebral disc. Interchanging radial distributions in annulus fibrosus. Biochem. J..

[44] Kielty C.M., Grant M.E., Royce P.M., Steinmann B. (2003). The Collagen Family: Structure, Assembly, and Organization in the Extracellular Matrix. Connective Tissue and Its Heritable Disorders: Molecular, Genetic, and Medical Aspects.

[45] Mouw J.K., Ou G., Weaver V.M. (2014). Extracellular matrix assembly: A multiscale deconstruction. Nat. Rev. Mol. Cell Biol..

[46] Yanagishita M. (1993). Function of proteoglycans in the extracellular matrix. Pathol. Int..

[47] Marchand F., Ahmed A.M. (1990). Investigation of the laminate structure of lumbar disc anulus fibrosus. Spine.

[48] Melrose J., Smith S.M., Appleyard R.C., Little C.B. (2008). Aggrecan, versican and type VI collagen are components of annular translamellar crossbridges in the intervertebral disc. Eur. Spine J..

[49] Hickey D.S., Hukins D.W.L. (1980). Relation between the Structure of the Annulus Fibrosus and the Function and Failure of the Intervertebral-Disk. Spine.

[50] Green T.P., Adams M.A., Dolan P. (1993). Tensile properties of the annulus fibrosus II. Ultimate tensile strength and fatigue life. Eur. Spine J..

[51] Yu J., Tirlapur U., Fairbank J., Handford P., Roberts S., Winlove C.P., Cui Z., Urban J. (2007). Microfibrils, elastin fibres and collagen fibres in the human intervertebral disc and bovine tail disc. J. Anat..

[52] Nerurkar N.L., Elliott D.M., Mauck R.L. (2010). Mechanical design criteria for intervertebral disc tissue engineering. J. Biomech..

[53] O’Connell G.D., Sen S., Elliott D.M. (2012). Human annulus fibrosus material properties from biaxial testing and constitutive modeling are altered with degeneration. Biomech. Model. Mechan..

[54] Ambard D., Cherblanc F. (2009). Mechanical behavior of annulus fibrosus: A microstructural model of fibers reorientation. Ann. Biomed. Eng..

[55] Best B.A., Guilak F., Setton L.A., Zhu W.B., Saednejad F., Ratcliffe A., Weidenbaum M., Mow V.C. (1994). Compressive Mechanical-Properties of the Human Anulus Fibrosus and Their Relationship to Biochemical-Composition. Spine.

[56] Perie D.S., MacLean J.J., Owen J.P., Iatridis J.C. (2006). Correlating material properties with tissue composition in enzymatically digested bovine annulus fibrosus and nucleus pulposus tissue. Ann. Biomed. Eng..

[57] Iatridis J.C., Setton L.A., Weidenbaum M., Mow V.C. (1997). Alterations in the mechanical behavior of the human lumbar nucleus pulposus with degeneration and aging. J. Orthop. Res..

[58] Trout J.J., Buckwalter J.A., Moore K.C. (1982). Ultrastructure of the human intervertebral disc: II. Cells of the nucleus pulposus. Anat. Rec..

[59] Bonetti M.I. (2009). Microfibrils: A cornerstone of extracellular matrix and a key to understand Marfan syndrome. Ital. J. Anat. Embryol..

[60] Akkiraju H., Nohe A. (2015). Role of Chondrocytes in Cartilage Formation, Progression of Osteoarthritis and Cartilage Regeneration. J. Dev. Biol..

[61] Muir H. (1995). The chondrocyte, architect of cartilage. Biomechanics, structure, function and molecular biology of cartilage matrix macromolecules. Bioessays.

[62] Calve J., Galland M. (1930). The intervertebral nucleus pulposus—Its anatomy, its physiology, its pathology. J. Bone Jt. Surg..

[63] Keyes D.C., Compere E.L. (1932). The normal and pathological physiology of the nucleus pulposus of the intervertebral disc—An anatomical, clinical, and experimental study. J. Bone Jt. Surg..

[64] Lotz J.C., Fields A.J., Liebenberg E.C. (2013). The Role of the Vertebral End Plate in Low Back Pain. Glob. Spine J..

[65] Moore R.J. (2006). The vertebral endplate: Disc degeneration, disc regeneration. Eur. Spine J..

[66] Mwale F., Roughley P., Antoniou J. (2004). Distinction between the extracellular matrix of the nucleus pulposus and hyaline cartilage: A requisite for tissue engineering of intervertebral disc. Eur. Cell Mater..

[67] Sophia Fox A.J., Bedi A., Rodeo S.A. (2009). The basic science of articular cartilage: Structure, composition, and function. Sports Health.

[68] Miller E.J., Rhodes R.K. (1982). Preparation and characterization of the different types of collagen. Methods Enzym..

[69] Kuhn K., Schmid T.M., Linsenmayer T.F., Rest M., Mayne R., Mayne R., Burgeson R.E. (1987). Structure and Function of Collagen Types.

[70] Grant J.P., Oxland T.R., Dvorak M.F. (2001). Mapping the structural properties of the lumbosacral vertebral endplates. Spine.

[71] Rodriguez A.G., Rodriguez-Soto A.E., Burghardt A.J., Berven S., Majumdar S., Lotz J.C. (2012). Morphology of the human vertebral endplate. J. Orthop. Res..

[72] Herkowitz H.N., Spine I.S.S.L. (2004). The Lumbar Spine.

[73] Nekkanty S., Yerramshetty J., Kim D.G., Zauel R., Johnson E., Cody D.D., Yeni Y.N. (2010). Stiffness of the endplate boundary layer and endplate surface topography are associated with brittleness of human whole vertebral bodies. Bone.

[74] Rudert M., Tillmann B. (1993). Lymph and Blood-Supply of the Human Intervertebral Disc—Cadaver Study of Correlations to Discitis. Acta Orthop. Scand..

[75] Nerlich A.G., Schaaf R., Walchli B., Boos N. (2007). Temporo-spatial distribution of blood vessels in human lumbar intervertebral discs. Eur. Spine J..

[76] Bogduk N., Tynan W., Wilson A.S. (1981). The Nerve Supply to the Human Lumbar Intervertebral Disks. J. Anat..

[77] Edgar M.A. (2007). The nerve supply of the lumbar intervertebral disc. J. Bone Jt. Surg. Br..

[78] Freemont A.J., Peacock T.E., Goupille P., Hoyland J.A., OBrien J., Jayson M.I.V. (1997). Nerve ingrowth into diseased intervertebral disc in chronic back pain. Lancet.

[79] Urban J.P.G., Roberts S. (2003). Degeneration of the intervertebral disc. Arthritis Res..

[80] Gaskin D.J., Richard P. (2011). Relieving Pain in America: A Blueprint for Transforming Prevention, Care, Education, and Research.

[81] Crow W.T., Willis D.R. (2009). Estimating cost of care for patients with acute low back pain: A retrospective review of patient records. J. Am. Osteopath. Assoc..

[82] Roberts S., Evans H., Trivedi J., Menage J. (2006). Histology and pathology of the human intervertebral disc. J. Bone Jt. Surg. Am..

[83] Battie M.C., Videman T., Levalahti E., Gill K., Kaprio J. (2008). Genetic and environmental effects on disc degeneration by phenotype and spinal level: A multivariate twin study. Spine.

[84] Buckwalter J.A. (1995). Aging and degeneration of the human intervertebral disc. Spine.

[85] Chan D., Song Y., Sham P., Cheung K.M. (2006). Genetics of disc degeneration. Eur. Spine J..

[86] Inoue N., Espinoza Orias A.A. (2011). Biomechanics of intervertebral disk degeneration. Orthop. Clin. North. Am..

[87] Pfirrmann C.W.A., Metzdorf A., Zanetti M., Hodler J., Boos N. (2001). Magnetic Resonance Classification of Lumbar Intervertebral Disc Degeneration. Spine.

[88] Radek M., Pacholczyk-Sienicka B., Jankowski S., Albrecht L., Grodzka M., Depta A., Radek A. (2016). Assessing the correlation between the degree of disc degeneration on the Pfirrmann scale and the metabolites identified in HR-MAS NMR spectroscopy. Magn. Reson. Imaging.

[89] Sinusas K. (2012). Osteoarthritis: Diagnosis and treatment. Am. Fam. Physician.

[90] Maetzel A., Li L.C., Pencharz J., Tomlinson G., Bombardier C., The Community Hypertension and Arthritis Project Study Team (2004). The economic burden associated with osteoarthritis, rheumatoid arthritis, and hypertension: A comparative study. Ann. Rheum. Dis..

[91] Goode A.P., Carey T.S., Jordan J.M. (2013). Low Back Pain and Lumbar Spine Osteoarthritis: How Are They Related?. Curr. Rheumatol. Rep..

[92] Dunlop R.B., Adams M.A., Hutton W.C. (1984). Disc space narrowing and the lumbar facet joints. J. Bone Jt. Surg. Br..

[93] Fujiwara A., Tamai K., An H.S., Kurihashi A., Lim T.H., Yoshida H., Saotome K. (2000). The relationship between disc degeneration, facet joint osteoarthritis, and stability of the degenerative lumbar spine. J. Spinal Disord..

[94] Fujiwara A., Lim T.H., An H.S., Tanaka N., Jeon C.H., Andersson G.B.J., Haughton V.M. (2000). The effect of disc degeneration and facet joint osteoarthritis on the segmental flexibility of the lumbar spine. Spine.

[95] Horkoff M., Maloon S. (2014). Dysphagia secondary to esophageal compression by cervical osteophytes: A case report. BCMJ.

[96] Milette P.C., Fontaine S., Lepanto L., Cardinal E., Breton G. (1999). Differentiating lumbar disc protrusions, disc bulges, and discs with normal contour but abnormal signal intensity. Magnetic resonance imaging with discographic correlations. Spine.

[97] Rim D.C. (2016). Quantitative Pfirrmann Disc Degeneration Grading System to Overcome the Limitation of Pfirrmann Disc Degeneration Grade. Korean J. Spine.

[98] Adams M.A., Hutton W.C. (1985). Gradual disc prolapse. Spine.

[99] Kortelainen P., Puranen J., Koivisto E., Lahde S. (1985). Symptoms and Signs of Sciatica and Their Relation to the Localization of the Lumbar-Disk Herniation. Spine.

[100] Frymoyer J.W. (1988). Back Pain and Sciatica. N. Engl. J. Med..

[101] Taher F., Essig D., Lebl D.R., Hughes A.P., Sama A.A., Cammisa F.P., Girardi F.P. (2012). Lumbar Degenerative Disc Disease: Current and Future Concepts of Diagnosis and Management. Adv. Orthop..

[102] Physical Therapist’s Guide to Degenerative Disc Disease. http://www.moveforwardpt.com/symptomsconditionsdetail.aspx?cid=514086b4-1272-4584-8742-ec6d2aa8f8cb.

[103] Nwuga V.C. (1983). Ultrasound in treatment of back pain resulting from prolapsed intervertebral disc. Arch. Phys. Med. Rehabil..

[104] Adams M.A., Stefanakis M., Dolan P. (2010). Healing of a painful intervertebral disc should not be confused with reversing disc degeneration: Implications for physical therapies for discogenic back pain. Clin. Biomech..

[105] Will Steroid Injections Help My Degenerative Disc Disease?. http://www.arksurgicalhospital.com/will-steroid-injections-help-my-degenerative-disc-disease/.

[106] Buttermann G.R. (2004). The effect of spinal steroid injections for degenerative disc disease. Spine J..

[107] Chou R., Huffman L.H. (2007). Medications for acute and chronic low back pain: A review of the evidence for an American Pain Society/American College of Physicians clinical practice guideline. Ann. Int. Med..

[108] Drugs, Medications, and Spinal Injections for Degenerative Disc Disease. https://www.spineuniverse.com/conditions/degenerative-disc/drugs-medications-spinal-injections-degenerative-disc-disease.

[109] Sluijter M.E., Cosman E.R. (1995). Method and Apparatus for Heating an Intervertebral Disc for Relief of Back Pain. U.S. Patent.

[110] Sluijter M.E., Cosman E.R. (1996). Thermal Denervation of an Intervertebral Disc for Relief of Back Pain. U.S. Patent.

[111] Sulaiman S.B., Keong T.K., Cheng C.H., Saim A.B., Idrus R.B. (2013). Tricalcium phosphate/hydroxyapatite (TCP-HA) bone scaffold as potential candidate for the formation of tissue engineered bone. Indian J. Med. Res..

[112] Spivak J.M., Hasharoni A. (2001). Use of hydroxyapatite in spine surgery. Eur. Spine J..

[113] Nouh M.R. (2012). Spinal fusion-hardware construct: Basic concepts and imaging review. World J. Radiol..

[114] Confusion about Spinal Fusion. https://www.spineuniverse.com/treatments/surgery/lumbar/confusion-about-spinal-fusion.

[115] Multilevel Spinal Fusion for Low Back Pain. https://www.spine-health.com/treatment/spinal-fusion/multilevel-spinal-fusion-low-back-pain.

[116] Quirno M., Goldstein J.A., Bendo J.A., Kim Y., Spivak J.M. (2011). The Incidence of Potential Candidates for Total Disc Replacement among Lumbar and Cervical Fusion Patient Populations. Asian Spine J..

[117] Deyo R.A., Nachemson A., Mirza S.K. (2004). Spinal-fusion surgery—The case for restraint. N. Engl. J. Med..

[118] Reeks J., Liang H. (2015). Materials and Their Failure Mechanisms in Total Disc Replacement. Lubricants.

[119] Serhan H., Mhatre D., Defossez H., Bono C.M. (2011). Motion-preserving technologies for degenerative lumbar spine: The past, present, and future horizons. Int. J. Spine Surg..

[120] Guterl C.C., See E.Y., Blanquer S.B., Pandit A., Ferguson S.J., Benneker L.M., Grijpma D.W., Sakai D., Eglin D., Alini M. (2013). Challenges and strategies in the repair of ruptured annulus fibrosus. Eur. Cell Mater..

[121] Bron J.L., Helder M.N., Meisel H.J., Van Royen B.J., Smit T.H. (2009). Repair, regenerative and supportive therapies of the annulus fibrosus: Achievements and challenges. Eur. Spine J..

[122] Vadala G., Mozetic P., Rainer A., Centola M., Loppini M., Trombetta M., Denaro V. (2012). Bioactive electrospun scaffold for annulus fibrosus repair and regeneration. Eur. Spine J..

[123] Cruz M.A., Hom W.W., DiStefano T.J., Merrill R., Torre O.M., Lin H.A., Hecht A.C., Illien-Junger S., Iatridis J.C. (2018). Cell-Seeded Adhesive Biomaterial for Repair of Annulus Fibrosus Defects in Intervertebral Discs. Tissue Eng. Part A.

[124] Alini M., Roughley P.J., Antoniou J., Stoll T., Aebi M. (2002). A biological approach to treating disc degeneration: Not for today, but maybe for tomorrow. Eur. Spine J..

[125] Sudo H., Minami A. (2011). Caspase 3 as a therapeutic target for regulation of intervertebral disc degeneration in rabbits. Arthritis Rheum..

[126] Sakai D., Mochida J., Iwashina T., Hiyama A., Omi H., Imai M., Nakai T., Ando K., Hotta T. (2006). Regenerative effects of transplanting mesenchymal stem cells embedded in atelocollagen to the degenerated intervertebral disc. Biomaterials.

[127] D’Este M., Eglin D., Alini M. (2018). Lessons to be learned and future directions for intervertebral disc biomaterials. Acta Biomater..

[128] Bowles R.D., Setton L.A. (2017). Biomaterials for intervertebral disc regeneration and repair. Biomaterials.

[129] Iu J., Massicotte E., Li S.Q., Hurtig M.B., Toyserkani E., Santerre J.P., Kandel R.A. (2017). In Vitro Generated Intervertebral Discs: Toward Engineering Tissue Integration. Tissue Eng. Part A.

[130] Yang J.C., Yang X.L., Wang L., Zhang W., Yu W.B., Wang N.X., Peng B.A., Zheng W.F., Yang G., Jiang X.Y. (2017). Biomimetic nanofibers can construct effective tissue-engineered intervertebral discs for therapeutic implantation. Nanoscale.

[131] Bhunia B.K., Kaplan D.L., Mandal B.B. (2018). Silk-based multilayered angle-ply annulus fibrosus construct to recapitulate form and function of the intervertebral disc. Proc. Natl. Acad. Sci. USA.

[132] Ghorbani M., Ai J., Nourani M.R., Azami M., Beni B.H., Asadpour S., Bordbar S. (2017). Injectable natural polymer compound for tissue engineering of intervertebral disc: In vitro study. Mater. Sci. Eng. C Mater..

[133] Gan Y., Li P., Wang L., Mo X., Song L., Xu Y., Zhao C., Ouyang B., Tu B., Luo L. (2017). An interpenetrating network-strengthened and toughened hydrogel that supports cell-based nucleus pulposus regeneration. Biomaterials.

[134] Halloran D.O., Grad S., Stoddart M., Dockery P., Alini M., Pandit A.S. (2008). An injectable cross-linked scaffold for nucleus pulposus regeneration. Biomaterials.

[135] Park S.-H., Cho H., Gil E.S., Mandal B.B., Min B.-H., Kaplan D.L. (2011). Silk-Fibrin/Hyaluronic Acid Composite Gels for Nucleus Pulposus Tissue Regeneration. Tissue Eng. Part A.

[136] Pereira D.R., Silva-Correia J., Oliveira J.M., Reis R.L., Pandit A., Biggs M.J. (2018). Nanocellulose reinforced gellan-gum hydrogels as potential biological substitutes for annulus fibrosus tissue regeneration. Nanomed. Nanotechol..

[137] Liu C., Zhu C., Li J., Zhou P., Chen M., Yang H., Li B. (2015). The effect of the fibre orientation of electrospun scaffolds on the matrix production of rabbit annulus fibrosus-derived stem cells. Bone Res..

[138] Pirvu T., Blanquer S.B.G., Benneker L.M., Grijpma D.W., Richards R.G., Alini M., Eglin D., Grad S., Li Z. (2015). A combined biomaterial and cellular approach for annulus fibrosus rupture repair. Biomaterials.

[139] Hu D., Wu D., Huang L., Jiao Y., Li L., Lu L., Zhou C. (2018). 3D bioprinting of cell-laden scaffolds for intervertebral disc regeneration. Mater. Lett..

[140] Yang F., Xiao D., Zhao Q., Chen Z., Liu K., Chen S., Sun X., Yue Q., Zhang R., Feng G. (2018). Fabrication of a novel whole tissue-engineered intervertebral disc for intervertebral disc regeneration in the porcine lumbar spine. RSC Adv..

[141] Choy A.T.H., Chan B.P. (2015). A Structurally and Functionally Biomimetic Biphasic Scaffold for Intervertebral Disc Tissue Engineering. PLoS One.

[142] Hudson K.D., Bonassar L.J. (2017). Hypoxic Expansion of Human Mesenchymal Stem Cells Enhances Three-Dimensional Maturation of Tissue-Engineered Intervertebral Discs. Tissue Eng. Part A.

[143] Hudson K.D., Mozia R.I., Bonassar L.J. (2015). Dose-Dependent Response of Tissue-Engineered Intervertebral Discs to Dynamic Unconfined Compressive Loading. Tissue Eng. Part A.

[144] Buckley C.T., Hoyland J.A., Fujii K., Pandit A., Iatridis J.C., Grad S. (2018). Critical aspects and challenges for intervertebral disc repair and regeneration—Harnessing advances in tissue engineering. JOR Spine.

